# Association of Sodium-Glucose Cotransporter 2 Inhibitors with Osteomyelitis and Other Lower Limb Safety Outcomes in Type 2 Diabetes Mellitus: A Systematic Review and Meta-Analysis of Randomised Controlled Trials

**DOI:** 10.3390/jcm12123958

**Published:** 2023-06-09

**Authors:** Alessandro Nani, Federica Carrara, Chiara Maria Eleonora Paulesu, Chiara Dalle Fratte, Matteo Padroni, Silvia Enisci, Maria Concetta Bilancio, Maria Silvia Romio, Federico Bertuzzi, Basilio Pintaudi

**Affiliations:** 1 Department of Medical Biotechnology and Translational Medicine, University of Milan, 20133 Milan, Italychiara.dallefratte@unimi.it (C.D.F.);; 2 Hospital Pharmacy, Humanitas Gavazzeni, 24125 Bergamo, Italy; 3 Department of Diabetology, Niguarda Hospital, 20162 Milan, Italy

**Keywords:** drug safety, lower limb complications, meta-analysis, osteomyelitis, SGLT2 inhibitors, type 2 diabetes mellitus

## Abstract

Our aim was to evaluate osteomyelitis and other major lower limb safety outcomes (i.e., peripheral artery disease or PAD, ulcers, atraumatic fractures, amputations, symmetric polyneuropathy, and infections) in patients affected by type 2 diabetes mellitus (T2DM) and treated with sodium-glucose cotransporter 2 inhibitors (SGLT2-is). We thus performed a systematic review and meta-analysis of randomised controlled trials (RCTs) comparing SGLT2-is at approved doses for T2DM with a placebo or standard of care. MEDLINE, Embase, and Cochrane CENTRAL were searched through August 2022. Separate intention-to-treat analyses were implemented for each molecule to calculate Mantel-Haenszel risk ratios (RR_MH_) with 95% confidence intervals (CIs) through a random-effects model. We processed data from 42 RCTs for a total of 29,491 and 23,052 patients, respectively assigned to SGLT2-i and comparator groups. SGLT2-is showed a pooled neutral effect on osteomyelitis, PAD, fractures, and symmetric polyneuropathy, whereas slightly deleterious sway on ulcers (RR_MH_ 1.39 [1.01–1.91]), amputations (RR_MH_ 1.27 [1.04–1.55]), and infections (RR_MH_ 1.20 [1.02–1.40]). In conclusion, SGLT2-is appear to not significantly interfere with the onset of osteomyelitis, PAD, lower limb fractures, or symmetric polyneuropathy, even though the number of these events proved consistently higher in the investigational groups; otherwise, local ulcers, amputations, and overall infections may be favoured by their employment. This study is registered with the Open Science Framework (OSF).

## 1. Introduction

Diabetes is a chronic multisystemic disease primarily compromising micro- and macrocirculation, bone mineral density, and host immune defence, especially when associated with other risk factors such as obesity, arterial hypertension, and dyslipidaemias. Despite recent significant progress in the comprehension of its pathophysiology and the consequent constant amelioration of prevention and treatment regimens, incidence and prevalence are still globally on the rise. In 2021, according to the World Development Indicators of the World Bank, 9.8% of the world population had diagnosed diabetes, of whom over 90% were type 2 cases.

Introduced over a decade ago, sodium-glucose cotransporter 2 inhibitors (SGLT2-is) have by now grown into a cornerstone of advanced anti-diabetic therapy. Their good safety profile [1,2,3], together with the varied therapeutic combinations with insulin and all the main oral anti-diabetic agents, has proved them useful for contrasting type 2 diabetes mellitus by promoting glycosuria. In clinical practice, SGLT2-is can indeed diminish both glycated haemoglobin by 0.6–1.2% (regardless of patient age and disease span) and cardiovascular risk (yet unconfirmed for ertugliflozin), fostering a concomitant reduction in blood pressure, body weight, and uricaemia [4,5,6,7,8]. Apparently, their cardiorenal protection is highly dependent on the glomerular filtration rate, for they exert their chief therapeutic activity on the SGLT2s located in the S1/S2 segments of preserved proximal convoluted tubules, where they can effectively hinder both sodium and glucose resorption [9,10,11]. Hypoglycaemias are rare and almost exclusively ascribable to the attendant employment of sulphonylureas or insulin [12,13,14], whereas urinary tract infections and genital mycoses are generally mild, easy to treat with standard antimicrobial agents, and rather prevalent over the first weeks of treatment (distinctly in women and the elderly, in the case of poor personal hygiene and dehydration) [15]. Further plausible adverse events may include euglycaemic diabetic ketoacidosis, accelerated osteoporosis, and an increased risk of peripheral artery disease (PAD), lower limb amputations, and fractures (singularly significant with canagliflozin), but appurtenant results from hitherto issued secondary studies are still inconclusive [7,16,17,18].

Osteomyelitis is typically a bacterial infection of the bone mediated by S. aureus (the commonest pathogen in both acute and chronic forms), coagulase-negative staphylococci, streptococci, and other less-represented bacteria. While acute onset is peculiar to either haematogenous seeding or direct inoculation (due to wound contamination through surgery or trauma), subacute and chronic infections suppose the contiguous spread from adjacent soft tissues and joints and are characteristic of patients with type 2 diabetes mellitus [19]. Although still widely underdiagnosed, the overall annual incidence of osteomyelitis has progressively risen over the past 50 years, with a doubling of diabetes-related cases to almost one-third of the total [19].

Given the complete lack, or rather, the limited availability of systematic analyses (however often contradictory or inconclusive) surveying the lower limb safety outcomes of SGLT2-is, in this review our aims are to establish whether this drug class plays a protective, neutral, or noxious role towards a so far unexplored event, i.e., osteomyelitis, and re-assess its effects on some of the other major diabetic complications affecting this body district.

## 2. Methods

This systematic review with meta-analysis was performed according to the Preferred Reporting Items for Systematic Reviews and Meta-Analyses (PRISMA) statement.

### 2.1. Search Strategy and Selection Criteria

From June to 15 August 2022, we implemented a thorough MEDLINE (PubMed), Embase, and Cochrane Library (CENTRAL) search harnessing the strings reported in the Supplementary Materials (Supplementary Table S1), which included all the following keywords: “Type 2 Diabetes Mellitus”, “Canagliflozin”, “Dapagliflozin”, “Empagliflozin”, “Ertugliflozin”, “Ipragliflozin”, “Luseogliflozin”, and “Tofogliflozin”; different filters were applied according to the database involved. Hence, we comprehended all randomised controlled trials (RCTs) executed on human patients affected by type 2 diabetes and exposed to SGLT2-is, provided that the following criteria were met: availability of the full-text article; exclusive investigation of adult (over 18 years old) non-pregnant patients; evidence of comparison between SGLT2-is at approved therapeutic dosage(s) for type 2 diabetes (intervention), with or without other anti-diabetic therapy, and placebo or standard of care (comparator); the presence of at least one of our selected outcomes, whether primary or secondary.

We excluded all the RCTs conducted on diabetes other than type 2 or published in languages diverse from English, and all the redundant entries from both PubMed and Embase searched through the Cochrane Central Register of Controlled Trials (CENTRAL), due to a less refined proprietary filter algorithm. Case reports, case series, commentaries, conference abstracts, cost-effectiveness analyses, editorials, letters, and studies with no comparator were rejected as well. Neither secondary studies nor study protocols (unless the latter provided relevant data for unedited papers) were included. No date restriction was applied. Some study authors were contacted to retrieve missing full-text articles and information.

**Figure 1:**
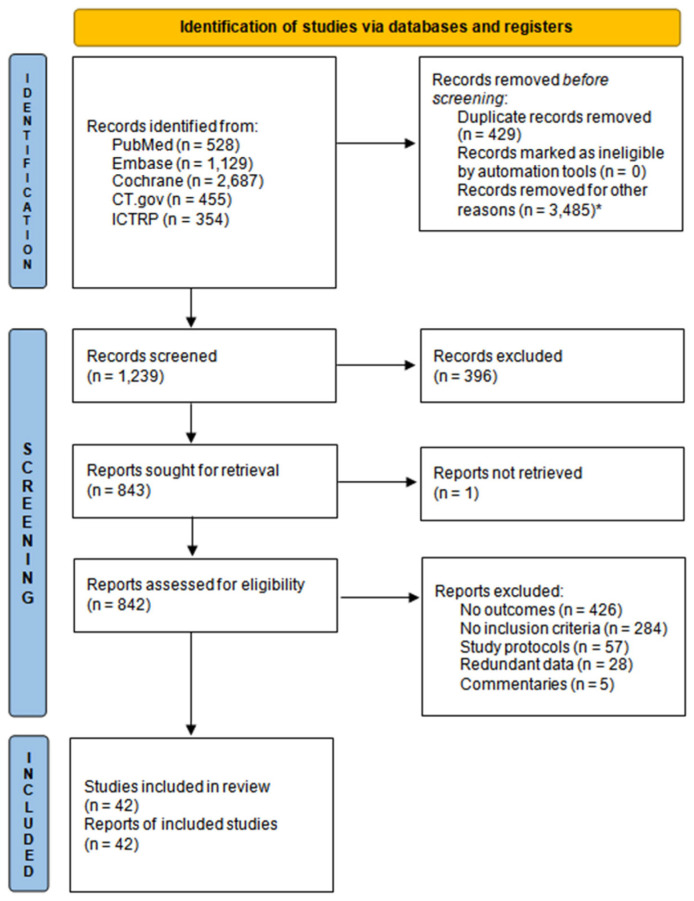
PRISMA flow diagram. * Cochrane CENTRAL redundant entries and CT.gov protocols of either already screened published articles or without outcomes of interest.

The selection process summarised in the PRISMA flow diagram (Figure 1) was carried out by three independent groups, and eventual conflicts were resolved by an external investigator (B.P.). Given the initial rather high number of screenable trials, the whole screening process was fulfilled through Rayyan—a web and mobile app for systematic reviews [20]—which streamlined our manual deduplication process by suggesting all possible duplicates.

### 2.2. Data Analysis

Data collection was independently accomplished by two authors (A.N. and B.P.), and possible conflicts were settled by internal discussion. Whenever available, summary estimates of the variables of interest were directly extracted just from the principal publications, otherwise suitably integrated with the possibly missing entries gathered from the results accompanying their protocols on online registries such as ClinicalTrials.gov, EU Clinical Trials Register, and UMIN-CTR; pertinent data from the intention-to-treat (ITT) population were always privileged. Further parameters/information collected were: first author, publication year, country, mean follow-up span, initial and in-between (only for crossover designs) washout periods, mean treatment extent, sample size, gender, ethnicity, average age, body mass index (BMI), obesity, smoke history, preliminary mean glycated haemoglobin, initial diabetic complications, background insulin therapy (with mean dosages), investigational drugs (with daily dosages) and comparators. All these data were reported in an electronic spreadsheet.

The major outcome considered was the incidence of osteomyelitis, a so far unexplored serious adverse event (SAE). For the sake of completeness and homology, we designated as secondary outcomes some of the main diabetic complications affecting the lower limbs: peripheral artery disease, lower limb ulcer(s), lower limb atraumatic fracture(s), lower limb amputation(s), symmetric polyneuropathy, and lower limb infections of each anatomical compartment. All the equivalent MedDRA terms accounting for our results were fully listed in Supplementary Tables S2–S5.

The risk of bias was gauged using the Cochrane risk of bias tool for RCTs on seven specific domains: random sequence generation, allocation concealment, blinding of participants and personnel, blinding of outcome assessment, incomplete outcome data, selective reporting, and overall bias. The results of these seven domains were consequently graded as either “low” risk of bias, “high” risk of bias, or “uncertain” risk of bias. The appraisal of bias risks was concomitantly achieved by two authors (B.P. and A.N.); possible conflicts were consequently fixed through consensus.

Mantel-Haenszel risk ratios (RR_MH_) with 95% confidence intervals (95% CI) were calculated for all the outcomes considered on an ITT basis, including also RCTs with zero events of interest (only when explicitly reported) and harnessing a random-effects model, independently of the heterogeneity level detected, as the validity of its values is limited in the case of a small number of component studies. Separate subgroup analyses were then performed for each outcome, pooling all the different SGLT2-i molecules at registered therapeutic doses. Likewise, given the rather long latency for most of them, every outcome was further analysed using only the related data from RCTs showing a mean follow-up span of at least 52 weeks. Study heterogeneity was evaluated thanks to the *I*^2^ statistics; whenever exceeding 50%, apposite sensitivity analyses were carried out. In order to estimate the existence of possible publication/disclosure biases, we examined the funnel plots generated for each outcome. The GRADE methodology was applied to rate the overall quality of the eligible RCTs for each outcome (Supplementary Table S7), utilising the GRADEpro GDT (GRADEpro Guideline Development Tool [Software], McMaster University and Evidence Prime, 2022, available from https://gradepro.org/, accessed on 21 November 2022).

Analyses were conducted through Review Manager (RevMan [Computer program], Version 5.4, The Cochrane Collaboration, 2020).

This work has been registered on the Open Science Framework (OSF, https://doi.org/10.17605/OSF.IO/PF8G5, accessed on 16 November 2022). Due to the secondary nature of already published data, institutional review board (IRB) approval and patient consent were superfluous.

## 3. Results

Figure 1 displays the PRISMA flow chart. In total, 42 trials were eventually included in our meta-analysis; their characteristics are summarised in Table 1. Gross study heterogeneity, measured by *I*^2^ tests, was mostly low with rare exceptions; results from each of the four sensitivity analyses executed for *I*^2^ values ≥ 50% were commensurable with their corresponding pooled RR_MH_ (Supplementary Figures S14–S17). The risk of bias assessment is shown beside every trial in all the forest plots; no publication bias was detected upon visual analysis of each funnel plot (Supplementary Figures S1–S7). Retrieved trials, respectively enrolled 29,491 and 23,052 patients in SGLT2-i and comparator groups, with a mean follow-up span of 50.9 weeks. At baseline, the mean age, HbA1c, and BMI of enrolled patients were 59.3 vs. 58.9 years, 64.8 vs. 65.0 mmol/mol, and 29.7 vs. 29.6 kg/m^2^, respectively for the two aforementioned subsets; moreover, Asian males (49.2 and 59.8%, respectively) were the most representative sample (Supplementary Table S6). Summary information on the event rates of every pre-specified outcome was systematically collected from all the published reports. Drug doses considered for all the investigational arms were canagliflozin 100–300 mg/day, dapagliflozin 10 mg/day, empagliflozin 10–25 mg/day, ertugliflozin 5–15 mg/day, ipragliflozin 25–50 mg/day, luseogliflozin 2.5–5 mg/day, and tofogliflozin 20 mg/day.

**Figure 2:**
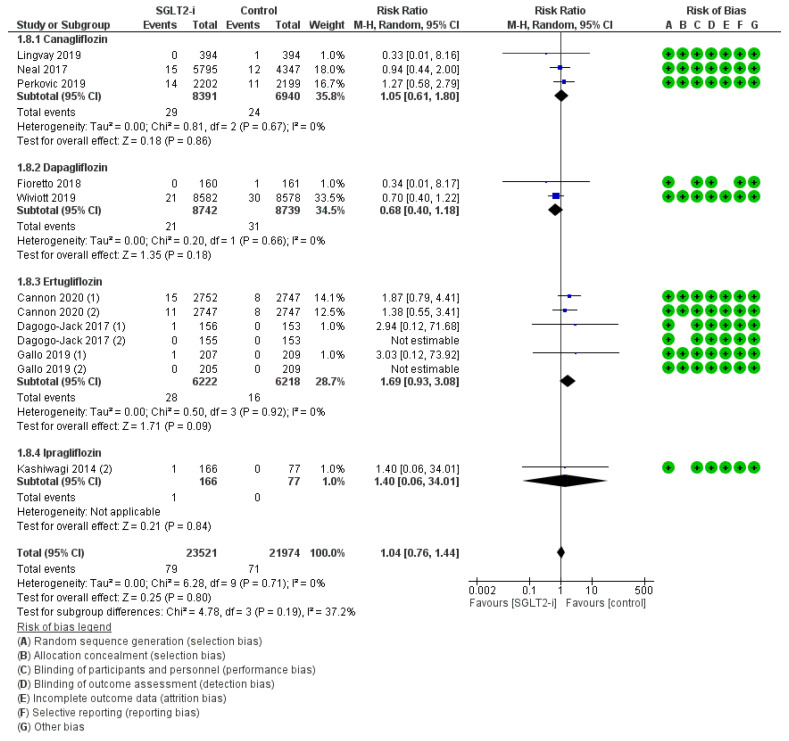
Overall analysis and molecular sub-analysis for osteomyelitis. SGLT2-i = sodium-glucose cotransporter 2 inhibitor(s); M-H = Mantel-Haenszel; CI = confidence interval [4,6,7,28,29,30,37,42,46].

**Figure 3:**
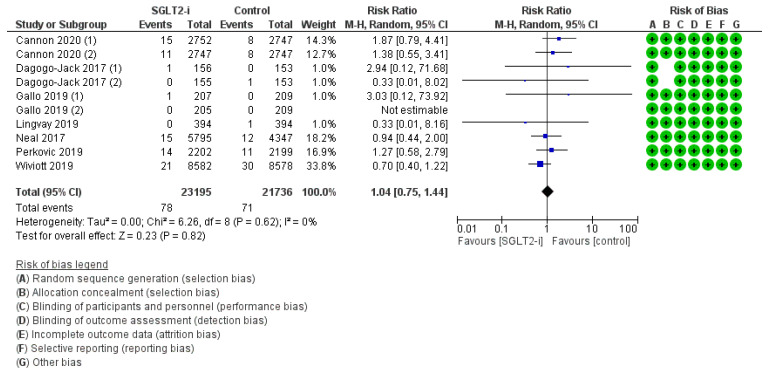
Follow-up span sub-analysis for osteomyelitis. SGLT2-i = sodium-glucose cotransporter 2 inhibitor(s); M-H = Mantel-Haenszel; CI = confidence interval [4,6,7,28,30,42,46].

Osteomyelitis was reported in 12 trials (3, 2, 6, and 1 with canagliflozin, dapagliflozin, ertugliflozin, and ipragliflozin, respectively) for a total of 79 events with SGLT2-is versus 71 events with comparators, thus resulting in a global RR_MH_ of 1.04 [0.76–1.44] (Figure 2). This neutral effect was confirmed in all sub-analyses, both by molecule (Canagliflozin: RR_MH_ 1.05 [0.61–1.80]; Dapagliflozin: RR_MH_ 0.68 [0.40–1.18]; Ertugliflozin: RR_MH_ 1.69 [0.93–3.08]; Ipragliflozin: RR_MH_ 1.40 [0.06–34.01]) and by follow-up span ≥ 52 weeks (RR_MH_ 1.04 [0.75–1.44]) (Figure 3).

**Figure 4:**
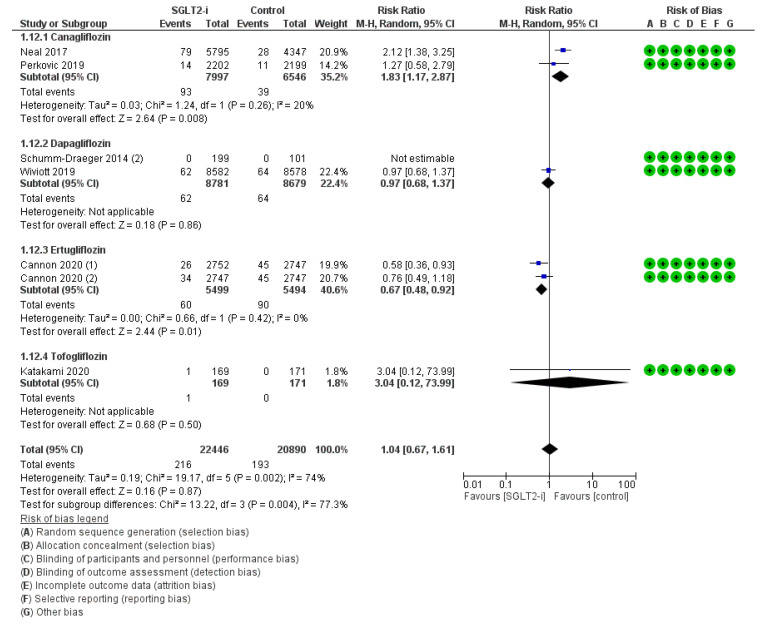
Overall analysis and molecular sub-analysis for peripheral artery disease. SGLT2-i = sodium-glucose cotransporter 2 inhibitor(s); M-H = Mantel-Haenszel; CI = confidence interval [4,6,7,38,46,52].

PAD was delineated in seven trials (2, 2, 2, and 1 with canagliflozin, dapagliflozin, ertugliflozin, and tofogliflozin, respectively) for an amount of 216 events with SGLT2-is versus 193 events with comparators, thus resulting in a pooled RR_MH_ of 1.04 [0.67–1.61] (Figure 4). This casual effect was only substantiated with Dapagliflozin (RR_MH_ 0.97 [0.68–1.37]) and Tofogliflozin (RR_MH_ 3.04 [0.12–73.99]), and in the follow-up span sub-analysis (RR_MH_ 1.04 [0.67–1.61]) (Supplementary Figure S8). Conversely, RR_MH_ was significantly increased for Canagliflozin (RR_MH_ 1.83 [1.17–2.87]), but slightly decreased for Ertugliflozin (RR_MH_ 0.67 [0.48–0.92]); the difference across molecules was statistically significant (*p* 0.004).

**Figure 5:**
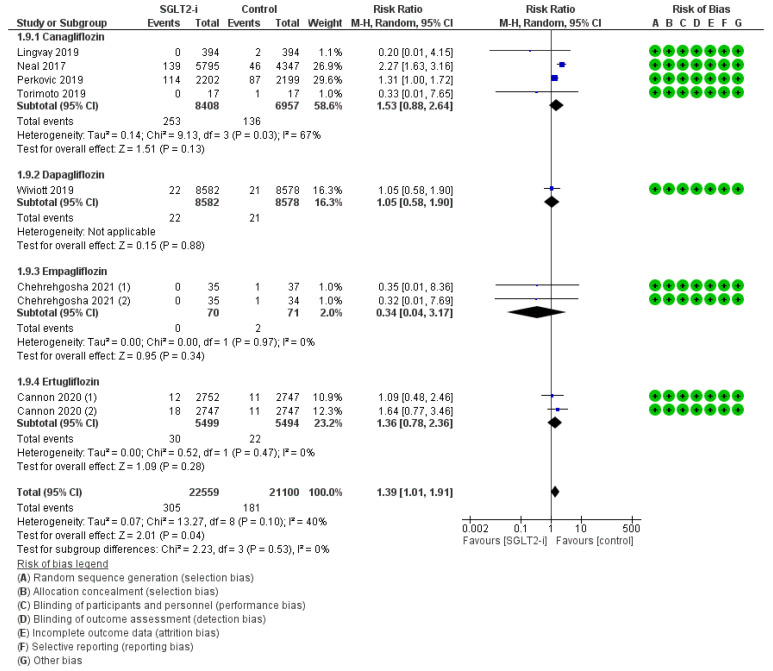
Overall analysis and molecular sub-analysis for lower limb ulcers. SGLT2-i = sodium-glucose cotransporter 2 inhibitor(s); M-H = Mantel-Haenszel; CI = confidence interval [4,6,7,27,42,46,56].

Lower limb ulcers were outlined in nine trials (4, 1, 2, and 2 with canagliflozin, dapagliflozin, empagliflozin, and ertugliflozin, respectively) for a total of 305 events with SGLT2-is versus 181 events with comparators, thus resulting in a global RR_MH_ of 1.39 [1.01–1.91] (Figure 5). Whilst concordant with the result of the relevant follow-up span sub-analysis (RR_MH_ 1.44 [1.03–2.01]) (Supplementary Figure S9), this marginally augmented risk was debunked by each molecule-based sub-analysis (Canagliflozin: RR_MH_ 1.53 [0.88–2.64]; Dapagliflozin: RR_MH_ 1.05 [0.58–1.90]; Empagliflozin: RR_MH_ 0.34 [0.04–3.17]; Ertugliflozin: RR_MH_ 1.36 [0.78–2.36]).

**Figure 6:**
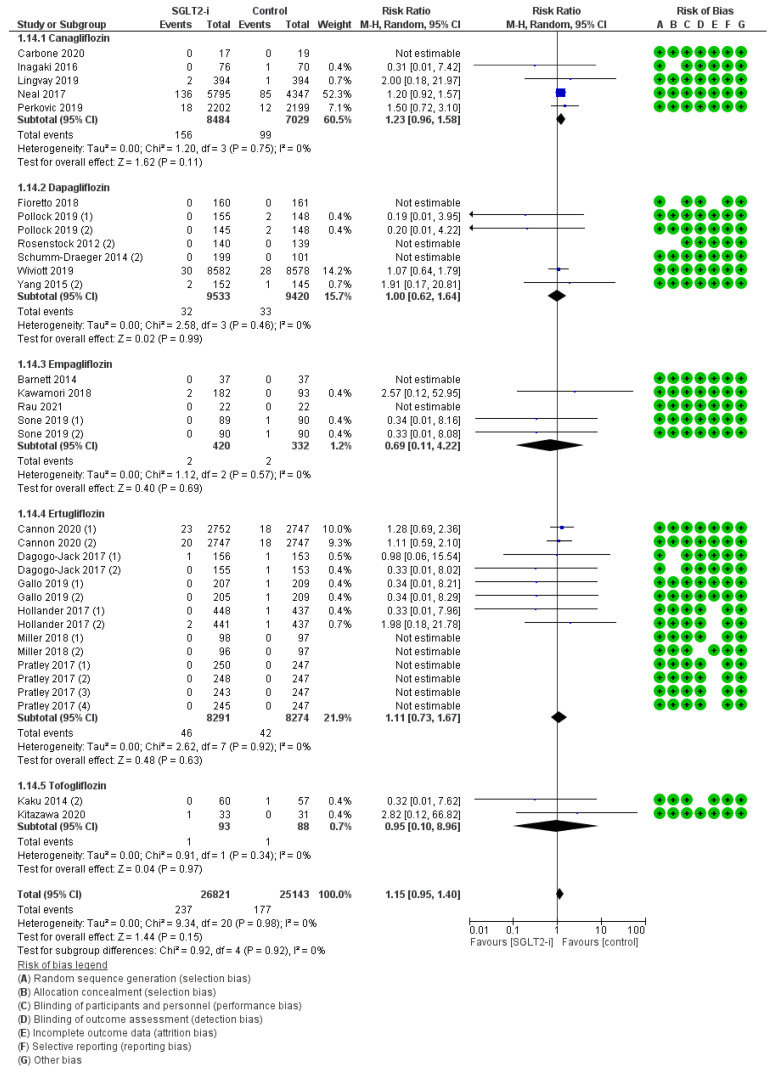
Overall analysis and molecular sub-analysis for lower limb fractures. SGLT2-i = sodium-glucose cotransporter 2 inhibitor(s); M-H = Mantel-Haenszel; CI = confidence interval [4,6,7,25,26,28,29,30,31,32,35,39,40,42,43,46,47,48,49,50,52,53,59].

Lower limb fractures were described in 33 trials (5, 7, 5, 14, and 2 with canagliflozin, dapagliflozin, empagliflozin, ertugliflozin, and tofogliflozin, respectively) for an amount of 237 events with SGLT2-is versus 177 events with comparators, thus resulting in a pooled RR_MH_ of 1.15 [0.95–1.40] (Figure 6). As for osteomyelitis, this incidental effect was confirmed in all sub-analyses, both by molecule (Canagliflozin: RR_MH_ 1.23 [0.96–1.58]; Dapagliflozin: RR_MH_ 1.00 [0.62–1.64]; Empagliflozin: RR_MH_ 0.69 [0.11–4.22]; Ertugliflozin: RR_MH_ 1.11 [0.73–1.67]; Tofogliflozin: RR_MH_ 0.95 [0.10–8.96]) and by follow-up span ≥ 52 weeks (RR_MH_ 1.17 [0.96–1.43]) (Supplementary Figure S10).

**Figure 7:**
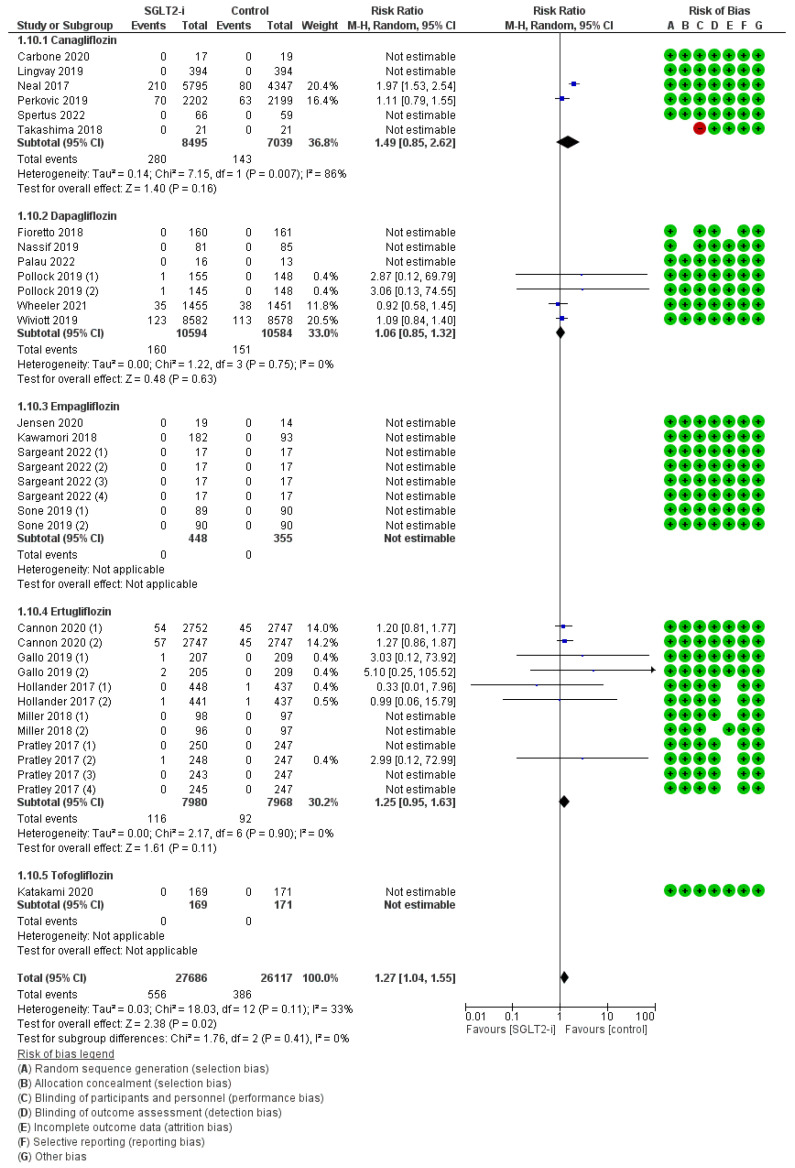
Overall analysis and molecular sub-analysis for amputations. SGLT2-i = sodium-glucose cotransporter 2 inhibitor(s); M-H = Mantel-Haenszel; CI = confidence interval [4,6,7,26,29,30,31,34,38,39,42,43,44,45,46,47,48,51,53,54,55,57].

Amputations were attested in 34 trials (6, 7, 8, 12, and 1 with canagliflozin, dapagliflozin, empagliflozin, ertugliflozin, and tofogliflozin, respectively) for a total of 556 events with SGLT2-is versus 386 events with comparators, thus resulting in a global RR_MH_ of 1.27 [1.04–1.55] (Figure 7). As already occurred for the ulcers, this mildly inflated risk, though also disclosed by the pertinent follow-up span sub-analysis (RR_MH_ 1.26 [1.02–1.56]) (Supplementary Figure S11), was confuted in every molecule-driven sub-analysis (Canagliflozin: RR 1.49 [0.85–2.62]; Dapagliflozin: RR_MH_ 1.06 [0.85–1.32]; Ertugliflozin: RR_MH_ 1.25 [0.95–1.63]; specific RRs uncomputable for Empagliflozin and Tofogliflozin due to the complete lack of events).

**Figure 8:**
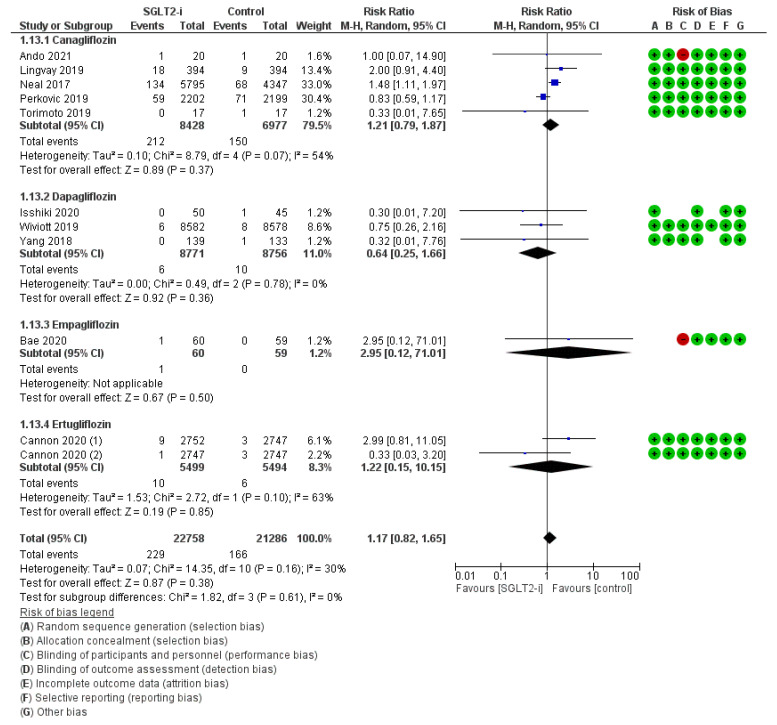
Overall analysis and molecular sub-analysis for symmetric polyneuropathy. SGLT2-i = sodium-glucose cotransporter 2 inhibitor(s); M-H = Mantel-Haenszel; CI = confidence interval [4,6,7,23,24,33,42,46,56,58].

Symmetric polyneuropathy was recorded in 11 trials (5, 3, 1, and 2 with canagliflozin, dapagliflozin, empagliflozin, and ertugliflozin, respectively) for an amount of 229 events with SGLT2-is versus 166 events with comparators, thus resulting in a pooled RR_MH_ of 1.17 [0.82–1.65] (Figure 8). As for osteomyelitis and fractures, this random effect was confirmed in all sub-analyses, both by molecule (Canagliflozin: RR_MH_ 1.21 [0.79–1.87]; Dapagliflozin: RR_MH_ 0.64 [0.25–1.66]; Empagliflozin: RR_MH_ 2.95 [0.12–71.01]; Ertugliflozin: RR_MH_ 1.22 [0.15–10.15]) and by follow-up span ≥ 52 weeks (RR_MH_ 1.22 [0.80–1.87]) (Supplementary Figure S12).

**Figure 9:**
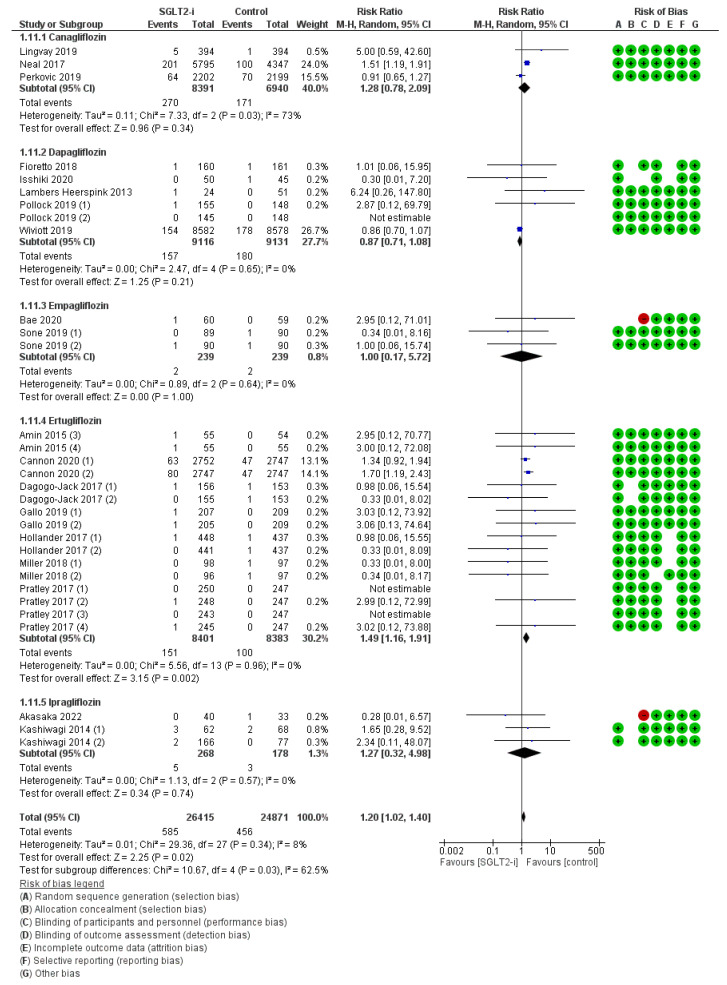
Overall analysis and molecular sub-analysis for lower limb infections. SGLT2-i = sodium-glucose cotransporter 2 inhibitor(s); M-H = Mantel-Haenszel; CI = confidence interval [4,6,7,21,22,24,28,29,30,31,33,36,37,41,42,43,46,47,48,53].

Lower limb infections, as the sum of all the infections affecting every single lower anatomical compartment (Supplementary Table S5), were detailed in 31 trials (3, 6, 3, 16, and 3 with canagliflozin, dapagliflozin, empagliflozin, ertugliflozin, and ipragliflozin, respectively) for a total of 585 events with SGLT2-is versus 456 events with comparators, thus resulting in a global RR_MH_ of 1.20 [1.02–1.40] (Figure 9). Albeit undermined both by the related follow-up span sub-analysis (RR_MH_ 1.22 [0.97–1.52]) (Supplementary Figure S13) and by some clashing molecular sub-analysis (Canagliflozin: RR_MH_ 1.28 [0.78–2.09]; Dapagliflozin: RR_MH_ 0.87 [0.71–1.08]; Empagliflozin RR_MH_ 1.00 [0.17–5.72]; Ipragliflozin: RR_MH_ 1.27 [0.32–4.98]), this datum of accrued risk was frankly validated with Ertugliflozin (RR_MH_ 1.49 [1.16–1.91]); the difference across molecules was statistically significant (*p* 0.03).

## 4. Discussion

Benefits from gliflozins have been by now acknowledged worldwide by all the leading international scientific societies [60,61,62]. This drug class has indeed revealed remarkable effects both in people affected by type 2 diabetes mellitus—by decreasing glycated haemoglobin and enhancing major metabolic parameters—and in cardiopathic or nephropathic patients, regardless of diabetes, where there is clear proof of cardio- and nephroprotective advantages [63]. In addition to all these assets, it was also highlighted a significant abatement of both all-cause and cardiovascular mortality, which allowed SGLT2-is to receive a privileged collocation in relevant therapeutic algorithms [64]. All this evidence, accrued from RCTs and observational studies based upon broad databases or populations, enables a larger-scale generalization of the efficacy of these drugs. Yet, it remains needful a constant parallel evaluation of their safety profile. In this regard, so far there has been plenty of real-world evidence supporting their decent safety, although the significant incidence of genitourinary tract infections demands carefulness whenever gliflozins are employed in common clinical scenarios [1,65]. Over the last few years, then, some warnings were also issued about possible adverse events affecting the lower limbs of subjects with type 2 diabetes, especially referring to an augmented risk of amputation [66,67]. This has consequently triggered a series of appraisals (mostly through secondary studies) which, despite their somewhat heterogeneous results, generally led to conclusions for the absence of an amplified risk, except for some subsets of patients and exquisitely for certain molecules from the class [18,68]. Nonetheless, it is still mandatory to gauge more thoroughly some of the other safety outcomes featured in admissible RCTs; in our case, some heretofore scantly explored events, i.e., lower limb ulcers and osteomyelitis or other infections affecting the same body district, represent all rapidly evolving situations which may be quite perilous for patients with type 2 diabetes. Hence, an added value of our systematic review is unquestionably embodied by the deeper knowledge of possible correlations between SGLT2-is and such potential risks to this population.

It is legitimate to make a direct comparison between our data and those in the literature only for a few of our outcomes, such as PAD and amputations. What has been so far published in this context is however exclusively limited to extended follow-up analyses [18,67,69]; otherwise, our work has also allowed us to highlight the hazards related to some early onset events. Particularly, after starting gliflozins, the risk ratio of lower limb infections, excluding osteomyelitides, is more pronounced (and statistically significant) in the pooled analysis, which incorporates trials with follow-up spans even inferior to six months; in fact, from a clinical perspective, infections generally constitute an acute complication, which is therefore likely to manifest itself precociously.

Among the outcomes explored by our research, it is also viable to hypothesise about some pathophysiological mechanisms for the ones which displayed an increased risk of development bound to gliflozin consumption, especially in the case of the coexistence of certain comorbidities (i.e., either local neuropathic or ischaemic alterations). Overall, individuals with type 2 diabetes mellitus exhibit a magnified ulcerative risk compared to the general population [70]: within the ulcer milieu, besides the noted higher inherent risk of infection, this process spreads far more easily to perilesional soft tissues and, in the most severe cases, to deeper bone structures. It follows an aggravated burden of amputations, both minor and major, which is independent of the primary lesion site. Several aetiopathological hypotheses have been sifted over the years to try to correlate the outset of such injuries of the lower limbs with a protracted gliflozin administration. Among the most accredited pathophysiological mechanisms now, there are already well-documented fluctuations of hematocrit and local vascular conditions [71]. As a clear example stands the exacerbated risk of mostly minor amputations, at first apparently associated with the exclusive employment of canagliflozin, where both the rise in blood viscosity (derived from haemoconcentration) and the relative hypovolemia induced by SGLT2-is may engender such a marked small blood vessel remodelling that could itself justify these data [7].

Further considerations are entailed by other outcomes, such as ulcers and infections, where a positive pathological anamnesis for either late-stage PAD or previous amputations constitutes an independent risk factor itself, alone sufficient to cause these events. In this regard, in our meta-analysis we reported data from RCTs with inclusion and exclusion criteria uneven as to these predisposing elements; hence, it ensued that the case mix of the enrolled population was also composed of secondary prevention subjects (often bearing pre-existent regional resections), who inevitably swayed, at least partially, risk ratio estimates. More limitations in our study could be attributed to both the heterogeneity of the measured outcome definitions (peculiarly the ones contemplating PAD, ulcers, diabetic neuropathy, and infections) and the frequent complete absence of their focussed external adjudication, which contributed to rife, albeit underestimated, detection and reporting biases. An effective, yet less practicable, expedient to remedy this criticality would consist in performing patient-level meta-analyses. Lastly, the clinical interpretation of discrepancies among data from some molecular sub-analyses demands a deeper enquiry, as they may expose proper peculiarities of certain gliflozins, unlike what has been hitherto reported in the literature.

## 5. Conclusions

In conclusion, SGLT2-is appear to not significantly interfere with the onset of osteomyelitis, PAD, lower limb fractures, or symmetric polyneuropathy, even though the number of these events proved consistently higher in almost all the investigational groups; otherwise, local ulcers, amputations, and overall infections may be favoured by their employment. These results underline how prior it is to carefully choose the optimum drug for each patient. All healthcare professionals should thus weigh the possible consequences elicited by an indiscriminate SGLT2-i prescription to subjects already affected by, or even at high risk of developing lower limb trophic lesions. These people would indeed appear more prone to rapidly worsening relapses. Despite the known advantages in terms of cardiovascular and renal protection, physicians should conveniently balance benefits and harms whenever they decide to either commence or pursue gliflozin therapy in patients more susceptible to lower limb complications. In light of such evidence, it would be appropriate if regulatory authorities and scientific societies explored more punctiliously any risk association, also through studies tailored to specific patient subgroups. Finally, a prompter pharmacovigilance network would definitely help clinicians and decision-makers to better judge the real extent of these phenomena.

## Figures and Tables

**Table 1:** Trials included in the meta-analysis. RR = risk ratio; CI = confidence interval; T2DM = type 2 diabetes mellitus; HF = heart failure; HFpEF = HF with preserved ejection fraction; NA = not available; CKD = chronic kidney disease; NE = not estimable; CV = cardiovascular; ACVD = atherosclerotic CV disease; HFrEF = HF with reduced ejection fraction; NAFLD = non-alcoholic fatty liver disease; BMI = body mass index; ERD = energy-restricted diet, i.e., −360 kcal/die; SGLT2-i = sodium-glucose cotransporter 2 inhibitor(s). ^1^ Study branches not included in the meta-analysis because not meeting all the inclusion criteria (i.e., administered SGLT2-i are not at approved therapeutic dosages for T2DM). ^2^ Higher dose available only for the last 12 weeks in the case of uncontrolled diabetes. ^3^ Risk ratio values not estimable because of the complete lack of related events. ^4^ Lower dose administered for the first 12 ± 2 weeks. ^5^ Patients with T2DM were only a subset of the overall study population. ^6^ Different doses were administered according to prolonged eGFR fluctuations over or under 60 mL/min per 1.73 m^2^.

	Population	Study Type	Dates	Intervention	Participants	Controls	Osteomyelitis RR (95% CI)	Peripheral Artery Disease RR (95% CI)	Ulcers RR (95% CI)	Fractures RR (95% CI)	Amputations RR (95% CI)	Symmetric Polineuropathy RR (95% CI)	Infections RR (95% CI)
Akasaka et al., 2022 [21]	Japanese adults with T2DM and HFpEF aged ≥20 years	Randomised controlled trial	2017–2019	Ipragliflozin 25 mg + Ipragliflozin50 mg	40	33	NA	NA	NA	NA	NA	NA	0.28 (0.01–6.57)
Amin et al., 2015 (1)^1^(2)^1^(3)(4)(5)^1^(6)^1^(7)^1^(8)^1^ [22]	Adults with T2DM aged 18–70 years	Randomised controlled trial	2010–2011	Ertugliflozin 1 mg; Ertugliflozin 1 mg; Ertugliflozin 5 mg; Ertugliflozin 5 mg; Ertugliflozin 10 mg; Ertugliflozin 10 mg; Ertugliflozin 25 mg; Ertugliflozin 25 mg	54 54 55 55 55 55 55 55	54 55 54 55 54 55 54 55	NA NA NA NA NA NA NA NA	NA NA NA NA NA NA NA NA	NA NA NA NA NA NA NA NA	NA NA NA NA NA NA NA NA	NA NA NA NA NA NA NA NA	NA NA NA NA NA NA NA NA	NA NA 2.95 (0.12–70.77) 3.00 (0.12–72.08) NA NA NA NA
Ando et al., 2021 [23]	Japanese adults with T2DM aged ≥20 years	Randomised controlled trial	2015–2018	Canagliflozin 100 mg	20	20	NA	NA	NA	NA	NA	1.00 (0.07–14.90)	NA
Bae et al., 2020 [24]	Korean adults with T2DM aged 19–80 years	Randomised controlled trial	2019–2020	Empagliflozin 10 mg + Empagliflozin25 mg^2^	60	59	NA	NA	NA	NA	NA	2.95 (0.12–71.01)	2.95 (0.12–71.01)
Barnett et al., 2014 [25]	Adults with T2DM and CKD aged ≥18 years	Randomised controlled trial	2010–2012	Empagliflozin 25 mg	37	37	NA	NA	NA	NE^3^	NA	NA	NA
Cannon et al., 2020 (1)(2) [4]	Adults with T2DM and ACVD aged ≥40 years	Randomised controlled trial	2013–2019	Ertugliflozin 5 mg; Ertugliflozin 15 mg	2752 2747	2747 2747	1.87 (0.79–4.41) 1.38 (0.55–3.41)	0.58 (0.36–0.93) 0.76 (0.49–1.18)	1.09 (0.48–2.46) 1.64 (0.77–3.46)	1.28 (0.69–2.36) 1.11 (0.59–2.10)	1.20 (0.81–1.77) 1.27 (0.86–1.87)	2.99 (0.81–11.05) 0.33 (0.03–3.20)	1.34 (0.92–1.94) 1.70 (1.19–2.43)
Carbone et al., 2020 [26]	Adults with T2DM and HFrEF aged ≥18 years	Randomised controlled trial	2016–2018	Canagliflozin 100 mg	17	19	NA	NA	NA	NE^3^	NE^3^	NA	NA
Chehrehgosha et al., 2021 (1)(2) [27]	Iranian adults with T2DM and NAFLD aged 20–65 years	Randomised controlled trial	2019–2020	Empagliflozin 10 mg; Empagliflozin 10 mg	35 35	37 34	NA NA	NA NA	0.35 (0.01–8.36) 0.32 (0.01–7.69)	NA NA	NA NA	NA NA	NA NA
Dagogo-Jack et al., 2017 (1)(2) [28]	Adults with T2DM aged ≥18 years	Randomised controlled trial	2014–2016	Ertugliflozin 5 mg; Ertugliflozin 15 mg	156 155	153 153	2.94 (0.12–71.68) NE^3^	NA NA	NA NA	0.98 (0.06–15.54) 0.33 (0.01–8.02)	NA NA	NA NA	0.98 (0.06–15.54) 0.33 (0.01–8.02)
Fioretto et al., 2018 [29]	Adults with T2DM and CKD 3A aged 18–74 years	Randomised controlled trial	2015–2017	Dapagliflozin 10 mg	160	161	0.34 (0.01–8.17)	NA	NA	NE^3^	NE^3^	NA	1.01 (0.06–15.95)
Gallo et al., 2019 (1)(2) [30]	Adults with T2DM aged ≥18 years	Randomised controlled trial	2013–2017	Ertugliflozin 5 mg; Ertugliflozin 15 mg	207 205	209 209	3.03 (0.12–73.92) NE^3^	NA NA	NA NA	0.34 (0.01–8.21) 0.34 (0.01-8.29)	3.03 (0.12-73.92) 5.10 (0.25–105.52)	NA NA	3.03 (0.12–73.92) 3.06 (0.13–74.64)
Hollander et al., 2017 (1)(2) [31]	Adults with T2DM aged ≥18 years	Randomised controlled trial	2013–2016	Ertugliflozin 5 mg; Ertugliflozin 15 mg	448 441	437 437	NA NA	NA NA	NA NA	0.33 (0.01–7.96) 1.98 (0.18–21.78)	0.33 (0.01-7.96) 0.99 (0.06–15.79)	NA NA	0.98 (0.06–15.55) 0.33 (0.01–8.09)
Inagaki et al., 2016 [32]	Japanese adults with T2DM aged ≥20 years	Randomised controlled trial	2014–2015	Canagliflozin 100 mg	76	70	NA	NA	NA	0.31 (0.01–7.42)	NA	NA	NA
Isshiki et al., 2020 [33]	Japanese adults with T2DM aged 20–74 years	Randomised controlled trial	2016–2019	Dapagliflozin 5 mg^4^ + Dapagliflozin10 mg	50	45	NA	NA	NA	NA	NA	0.30 (0.01–7.20)	0.30 (0.01–7.20)
Jensen et al., 2020 [34]	Adults with HFrEF aged 18–84 years^5^	Randomised controlled trial	2017–2020	Empagliflozin 10 mg	19	14	NA	NA	NA	NA	NE^3^	NA	NA
Kaku et al., 2014 (1)^1^(2)(3)^1^ [35]	Japanese adults with T2DM aged 20–74 years	Randomised controlled trial	2010–2012	Tofogliflozin 10 mg; Tofogliflozin 20 mg; Tofogliflozin 40 mg	59 60 59	57 57 57	NA NA NA	NA NA NA	NA NA NA	NA 0.32 (0.01–7.62) NA	NA NA NA	NA NA NA	NA NA NA
Kashiwagi et al., 2014 (1) [36]	Japanese adults with T2DM aged ≥20 years	Randomised controlled trial	2010	Ipragliflozin 50 mg	62	68	NA	NA	NA	NA	NA	NA	1.65 (0.28–9.52)
Kashiwagi et al., 2014 (2) [37]	Japanese adults with T2DM aged ≥20 years	Randomised controlled trial	2010	Ipragliflozin 50 mg	166	77	1.40 (0.06–34.01)	NA	NA	NA	NA	NA	2.34 (0.11–48.07)
Katakami et al., 2020 [38]	Japanese adults with T2DM aged 30–74 years	Randomised controlled trial	2016–2019	Tofogliflozin 20 mg	169	171	NA	3.04 (0.12–73.99)	NA	NA	NE^3^	NA	NA
Kawamori et al., 2018 [39]	Japanese adults with T2DM aged ≥20 years	Randomised controlled trial	2015–2017	Empagliflozin 10 mg + Empagliflozin25 mg	182	93	NA	NA	NA	2.57 (0.12–52.95)	NE^3^	NA	NA
Kitazawa et al., 2020 [40]	Japanese adults with T2DM aged 20–74 years	Randomised controlled trial	2017–2018	Tofogliflozin 20 mg	33	31	NA	NA	NA	2.82 (0.12–66.82)	NA	NA	NA
Lambers Heerspink et al., 2013 [41]	Adults with T2DM aged 18–70 years	Randomised controlled trial	2009–2010	Dapagliflozin 10 mg	24	51	NA	NA	NA	NA	NA	NA	6.24 (0.26–147.80)
Lingvay et al., 2019 [42]	Adults with T2DM aged ≥18 years	Randomised controlled trial	2017–2018	Canagliflozin 300 mg^6^ + Canagliflozin 100 mg^6^	394	394	0.33 (0.01–8.16)	NA	0.20 (0.01–4.15)	2.00 (0.18–21.97)	NE^3^	2.00 (0.91–4.40)	5.00 (0.59–42.60)
Miller et al., 2018 (1)(2) [43]	Adults with T2DM aged ≥18 years	Randomised controlled trial	2014–2016	Ertugliflozin 5 mg + Sitagliptin 100 mg; Ertugliflozin 15 mg + Sitagliptin 100 mg	98 96	97 97	NA NA	NA NA	NA NA	NE^3^ NE^3^	NE^3^ NE^3^	NA NA	0.33 (0.01–8.00) 0.34 (0.01–8.17)
Nassif et al., 2019 [44]	Adults with HFrEF aged >18^5^	Randomised controlled trial	2016–2019	Dapagliflozin 10 mg	81	85	NA	NA	NA	NA	NE^3^	NA	NA
Neal et al., 2017 [7]	Adults with T2DM and ACVD aged ≥30 years OR Adults with T2DM and ≥2 CV risk factors aged ≥50 years	Randomised controlled trial	2009–2017	Canagliflozin 100 mg + Canagliflozin 300 mg	5795	4347	0.94 (0.44–2.00)	2.12 (1.38–3.25)	2.27 (1.63–3.16)	1.20 (0.92–1.57)	1.97 (1.53–2.54)	1.48 (1.11–1.97)	1.51 (1.19–1.91)
Palau et al., 2022 [45]	Adults with HFrEF aged >18 years^5^	Randomised controlled trial	2019–2021	Dapagliflozin 10 mg	16	13	NA	NA	NA	NA	NE^3^	NA	NA
Perkovic et al., 2019 [46]	Adults with T2DM and CKD aged ≥30 years	Randomised controlled trial	2014–2018	Canagliflozin 100 mg	2202	2199	1.27 (0.58–2.79)	1.27 (0.58–2.79)	1.31 (1.00–1.72)	1.50 (0.72–3.10)	1.11 (0.79–1.55)	0.83 (0.59–1.17)	0.91 (0.65–1.27)
Pollock et al., 2019 (1)(2) [47]	Adults with T2DM and CKD aged ≥18 years	Randomised controlled trial	2015–2018	Dapagliflozin 10 mg + Saxagliptin 2.5 mg; Dapagliflozin 10 mg	155 145	148 148	NA NA	NA NA	NA NA	0.19 (0.01–3.95) 0.20 (0.01–4.22)	2.87 (0.12–69.79) 3.06 (0.13–74.55)	NA NA	2.87 (0.12–69.79) NE^3^
Pratley et al., 2017 (1)(2)(3)(4) [48]	Adults with T2DM aged ≥18 years	Randomised controlled trial	2014–2016	Ertugliflozin 5 mg; Ertugliflozin 15 mg; Ertugliflozin 5 mg + Sitagliptin 100 mg; Ertugliflozin 15 mg + Sitagliptin 100 mg	250 248 243 245	247 247 247 247	NA NA NA NA	NA NA NA NA	NA NA NA NA	NE^3^ NE^3^ NE^3^ NE^3^	NE^3^ 2.99 (0.12–72.99) NE^3^ NE^3^	NA NA NA NA	NE^3^ 2.99 (0.12–72.99) NE^3^ 3.02 (0.12–73.88)
Rau et al., 2021 [49]	Adults with T2DM aged 18–84 years	Randomised controlled trial	2017–2019	Empagliflozin 10 mg	22	22	NA	NA	NA	NE^3^	NA	NA	NA
Rosenstock et al., 2012 (1)^1^(2) [50]	Adults with T2DM aged ≥18 years	Randomised controlled trial	2008–2010	Dapagliflozin 5 mg; Dapagliflozin 10 mg	141 140	139 139	NA NA	NA NA	NA NA	NA NE^3^	NA NA	NA NA	NA NA
Sargeant et al., 2022 (1)(2)(3)(4) [51]	Adults with T2DM and BMI ≥25 kg/m^2^ aged 30–75 years	Randomised controlled trial	2017–2019	Empagliflozin 25 mg; Empagliflozin 25 mg; Empagliflozin 25 mg + ERD; Empagliflozin 25 mg + ERD	17 17 17 17	17 17 17 17	NA NA NA NA	NA NA NA NA	NA NA NA NA	NA NA NA NA	NE^3^ NE^3^ NE^3^ NE^3^	NA NA NA NA	NA NA NA NA
Schumm-Draeger et al., 2014 (1)^1^(2) [52]	Adults with T2DM aged 18–77 years	Randomised controlled trial	2010–2011	Dapagliflozin 5 mg; Dapagliflozin 10 mg	100 199	101 101	NA NA	NA NE^3^	NA NA	NA NE^3^	NA NA	NA NA	NA NA
Sone et al., 2019 (1)(2) [53]	Japanese adults with T2DM aged 20–74 years	Randomised controlled trial	2015–2018	Empagliflozin 10 mg; Empagliflozin 25 mg	89 90	90 90	NA NA	NA NA	NA NA	0.34 (0.01–8.16) 0.33 (0.01–8.08)	NE^3^ NE^3^	NA NA	0.34 (0.01–8.16) 1.00 (0.06–15.74)
Spertus et al., 2022 [54]	Adults with HF aged ≥18 years^5^	Randomised controlled trial	2020–2021	Canagliflozin 100 mg	66	59	NA	NA	NA	NA	NE^3^	NA	NA
Takashima et al., 2018 [55]	Japanese adults with T2DM and CKD aged 20–80 years	Randomised controlled trial	2016–2017	Canagliflozin 100 mg	21	21	NA	NA	NA	NA	NE^3^	NA	NA
Torimoto et al., 2019 [56]	Japanese adults with T2DM aged 18–79 years	Randomised controlled trial	2015–2018	Canagliflozin 100 mg	17	17	NA	NA	0.33 (0.01–7.65)	NA	NA	0.33 (0.01–7.65)	NA
Wheeler et al., 2021 [57]	Adults with proteinuric CKD aged ≥18 years^5^	Randomised controlled trial	2017–2020	Dapagliflozin 10 mg	1455	1451	NA	NA	NA	NA	0.92 (0.58–1.45)	NA	NA
Wiviott et al., 2019 [6]	Adults with T2DM and ACVD or multiple CV risk factors aged ≥40 years OR Men with T2DM and ≥1 CV risk factor aged ≥55 years OR Women with T2DM and ≥1 CV risk factor aged ≥60 years	Randomised controlled trial	2013–2018	Dapagliflozin 10 mg	8582	8578	0.70 (0.40–1.22)	0.97 (0.68–1.37)	1.05 (0.58–1.90)	1.07 (0.64–1.79)	1.09 (0.84–1.40)	0.75 (0.26–2.16)	0.86 (0.70–1.07)
Yang et al., 2018 [58]	Asian adults with T2DM aged ≥18 years	Randomised controlled trial	2014–2016	Dapagliflozin 10 mg	139	133	NA	NA	NA	NA	NA	0.32 (0.01–7.76)	NA
Yang et al., 2015 (1)^1^(2) [59]	Asian adults with T2DM aged ≥18 years	Randomised controlled trial	2010–2013	Dapagliflozin 5 mg; Dapagliflozin 10 mg	147 152	145 145	NA NA	NA NA	NA NA	NA 1.91 (0.17–20.81)	NA NA	NA NA	NA NA

## Data Availability

The datasets generated and/or analysed during the current study are available from the corresponding author upon reasonable request.

## Supplementary Materials

The following supporting information can be downloaded at: https://www.mdpi.com/article/10.3390/jcm12123958/s1, Figure S1: Funnel plot for osteomyelitis. Figure S2: Funnel plot for peripheral artery disease. Figure S3: Funnel plot for lower limb ulcers. Figure S4: Funnel plot for lower limb fractures. Figure S5: Funnel plot for lower limb amputations. Figure S6: Funnel plot for symmetric polyneuropathy. Figure S7: Funnel plot for lower limb infections. Figure S8: Follow-up span sub-analysis for peripheral artery disease. Figure S9: Follow-up span sub-analysis for lower limb ulcers. Figure S10: Follow-up span sub-analysis for lower limb fractures. Figure S11: Follow-up span sub-analysis for lower limb amputations. Figure S12: Follow-up span sub-analysis for symmetric polyneuropathy. Figure S13: Follow-up span sub-analysis for lower limb infections. Figure S14: Sensitivity analysis for overall peripheral artery disease. Figure S15: Sensitivity analysis for peripheral artery disease with a follow-up ≥ 52 weeks. Figure S16: Sensitivity analysis for lower limb ulcers with a follow-up ≥ 52 weeks. Figure S17: Sensitivity analysis for symmetric polyneuropathy with a follow-up ≥ 52 weeks. Table S1: Database query strings and filters. Table S2: MedDRA terms for peripheral artery disease. Table S3: MedDRA terms for lower limb ulcers. Table S4: MedDRA terms for symmetric polyneuropathy. Table S5: MedDRA terms for lower limb infections. Table S6: Baseline characteristics. Table S7: GRADE summary.
