# Supplementary material for: Association of Sodium-Glucose Cotransporter 2 Inhibitors with Osteomyelitis and Other Lower Limb Safety Outcomes in Type 2 Diabetes Mellitus: A Systematic Review and Meta-Analysis of Randomised Controlled Trials
Source: J Clin Med. 2023 Jun 9;12(12):3958. doi: 10.3390/jcm12123958 (PMC10299360; doi:10.3390/jcm12123958)
Supplement: Supplementary file 1 [file jcm-12-03958-s001.zip › jcm-2399757-supplementary.pdf]

SUPPLEMENTARY MATERIALS

Figure S1: Funnel plot for osteomyelitis.

Figure S2: Funnel plot for peripheral artery disease.

Figure S3: Funnel plot for lower limb ulcers.

Figure S4: Funnel plot for lower limb fractures.

Figure S5: Funnel plot for lower limb amputations.

Figure S6: Funnel plot for symmetric polyneuropathy.

Figure S7: Funnel plot for lower limb infections.

Figure S8: Follow-up span sub-analysis for peripheral artery disease.

Figure S9: Follow-up span sub-analysis for lower limb ulcers.

Figure S10: Follow-up span sub-analysis for lower limb fractures.

Figure S11: Follow-up span sub-analysis for lower limb amputations.

Figure S12: Follow-up span sub-analysis for symmetric polyneuropathy.

Figure S13: Follow-up span sub-analysis for lower limb infections.

Figure S14: Sensitivity analysis for overall peripheral artery disease.

Figure S15: Sensitivity analysis for peripheral artery disease with a follow-up ≥ 52 weeks.

Figure S16: Sensitivity analysis for lower limb ulcers with a follow-up ≥ 52 weeks.

Figure S17: Sensitivity analysis for symmetric polyneuropathy with a follow-up ≥ 52 weeks.

Table S1: Database query strings and filters.

Table S2: MedDRA terms for peripheral artery disease.

Table S3: MedDRA terms for lower limb ulcers.

Table S4: MedDRA terms for symmetric polyneuropathy.

Table S5: MedDRA terms for lower limb infections.

Table S6: Baseline characteristics.

Table S7: GRADE summary.

Figure S1: Funnel plot for osteomyelitis.

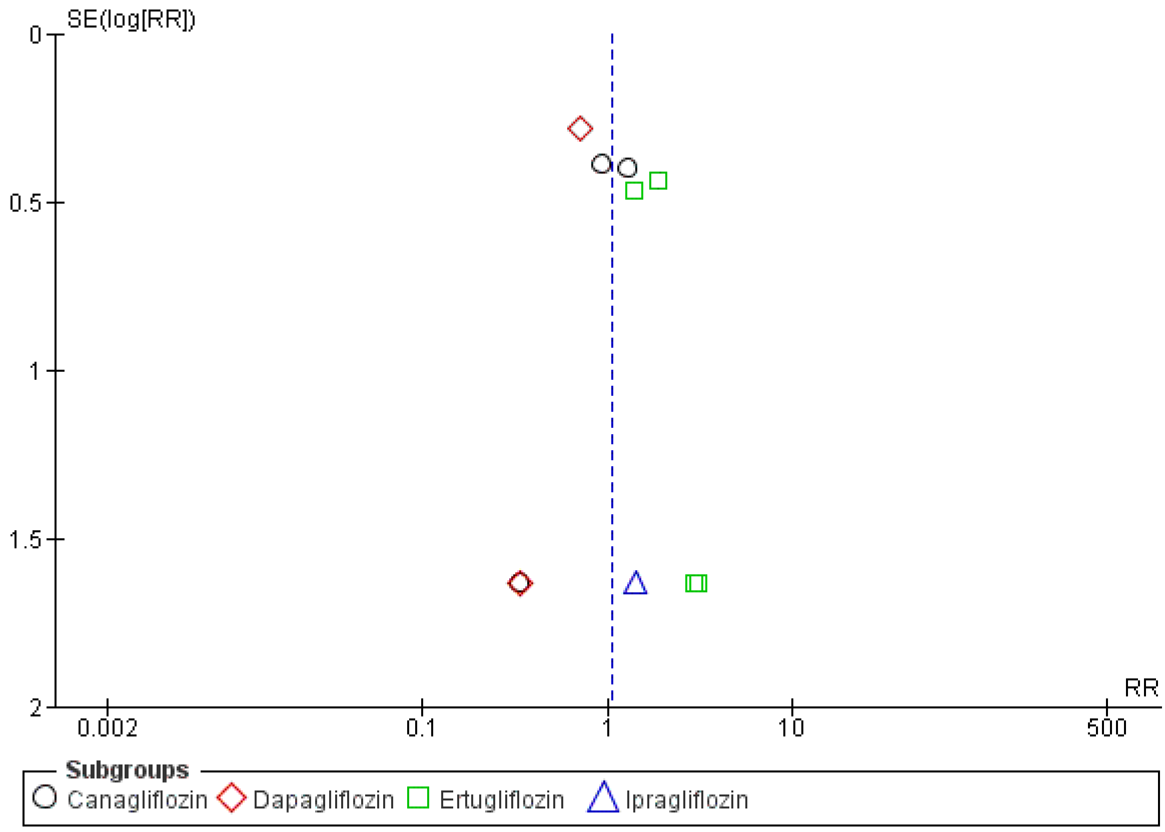

SE, standard error; RR, risk ratio.

Figure S2: Funnel plot for peripheral artery disease.

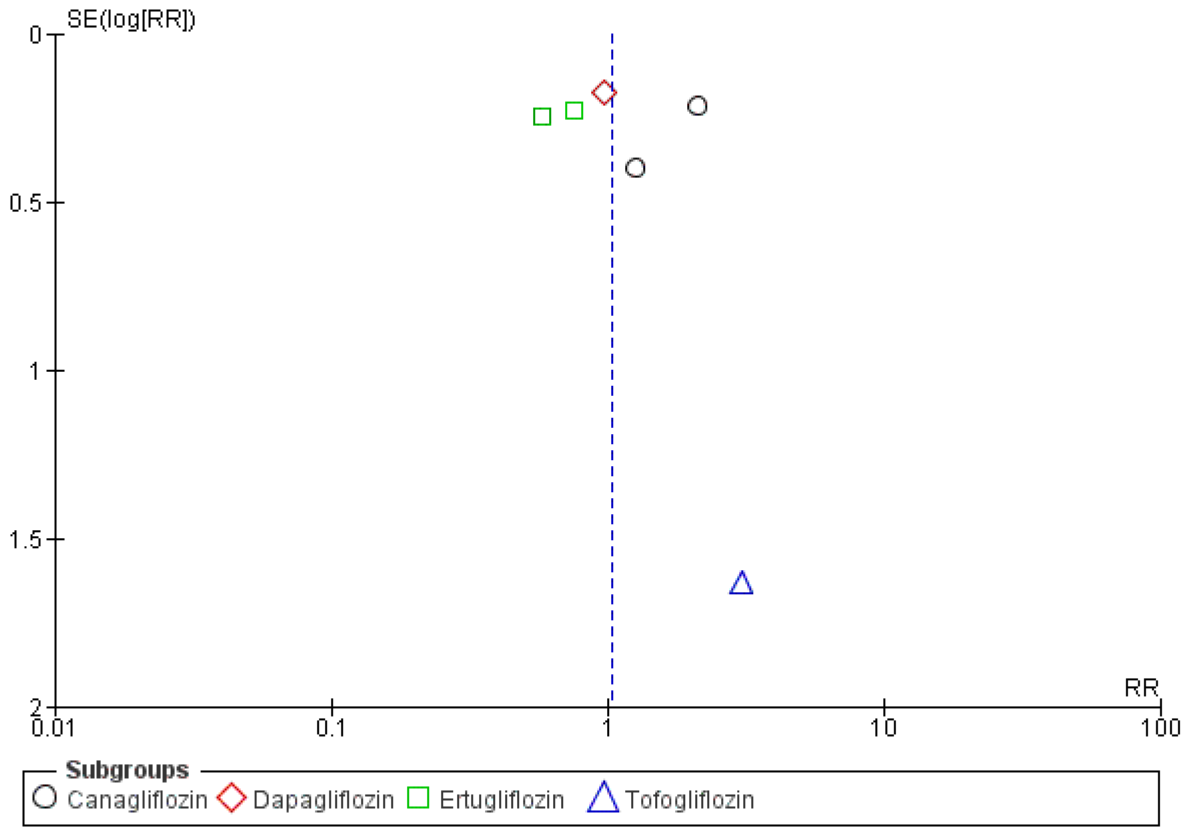

SE, standard error; RR, risk ratio.

Figure S3: Funnel plot for lower limb ulcers.

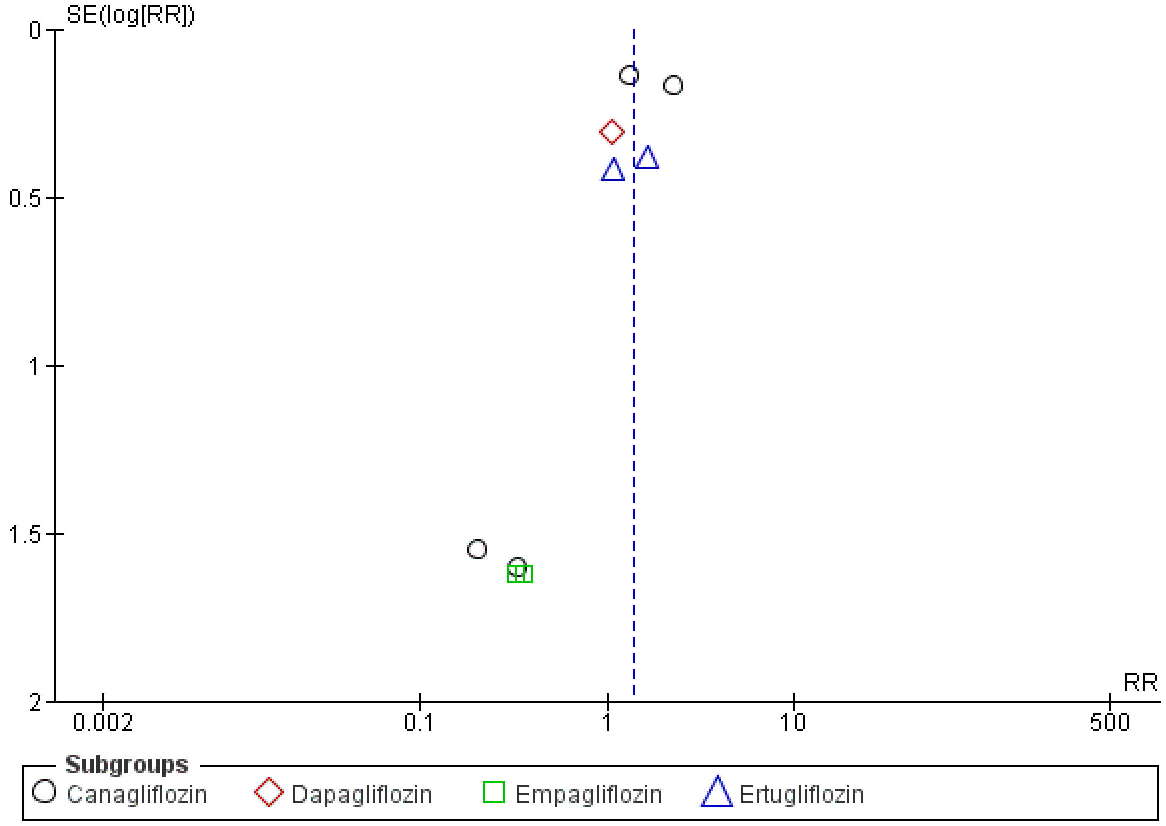

SE, standard error; RR, risk ratio.

Figure S4: Funnel plot for lower limb fractures.

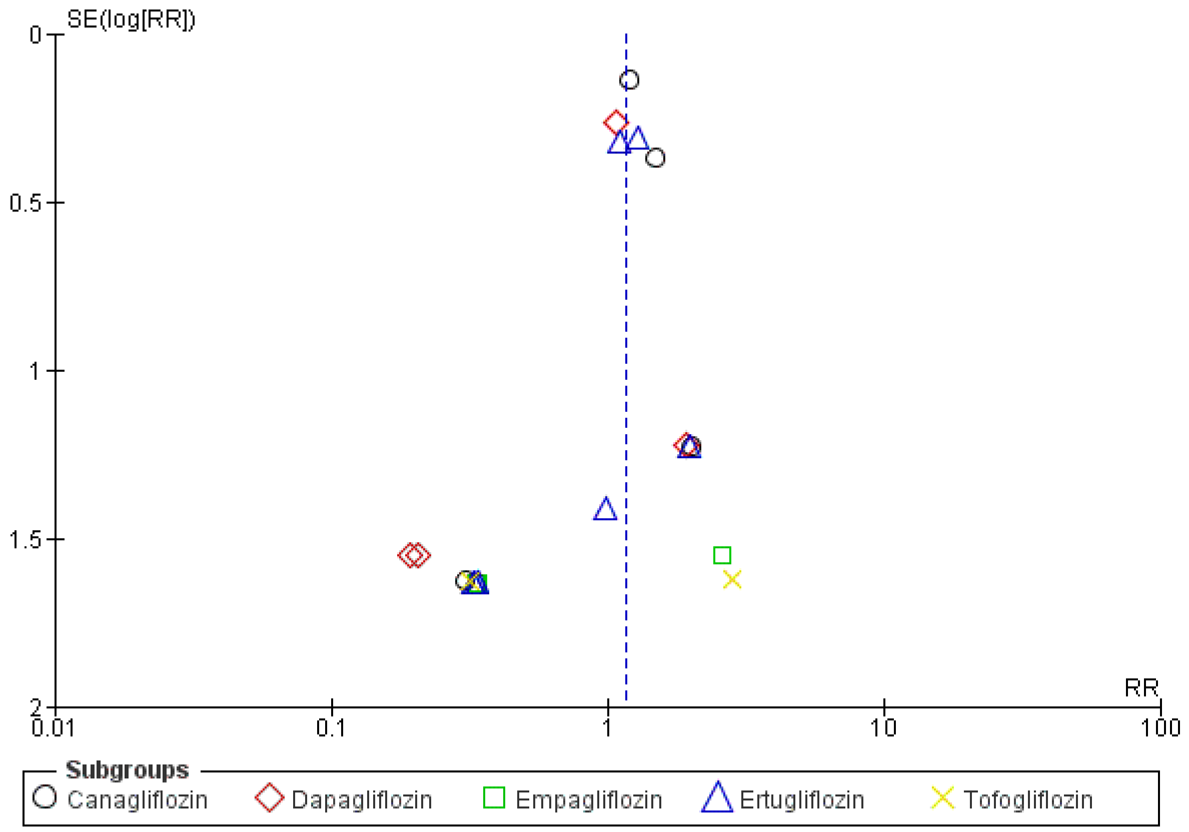

SE, standard error; RR, risk ratio.

Figure S5: Funnel plot for lower limb amputations.

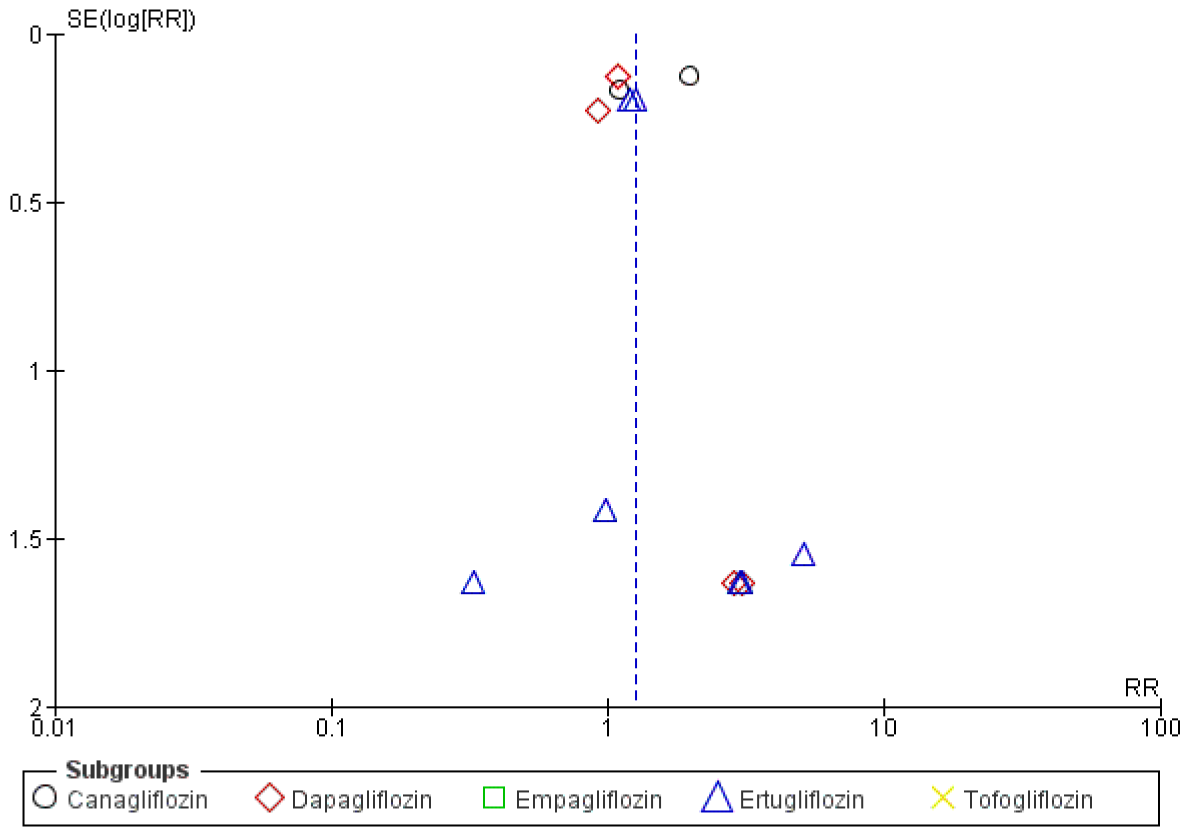

SE, standard error; RR, risk ratio.

Figure S6: Funnel plot for symmetric polyneuropathy.

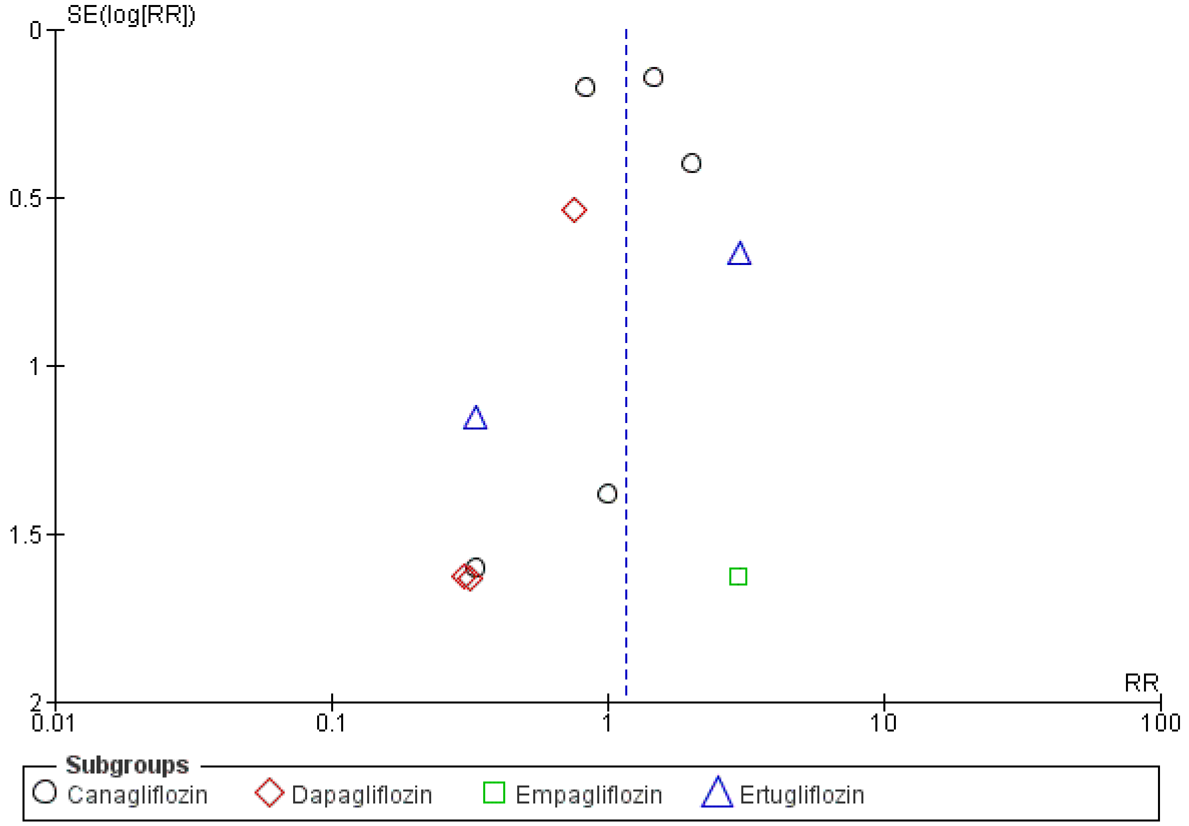

SE, standard error; RR, risk ratio.

Figure S7: Funnel plot for lower limb infections.

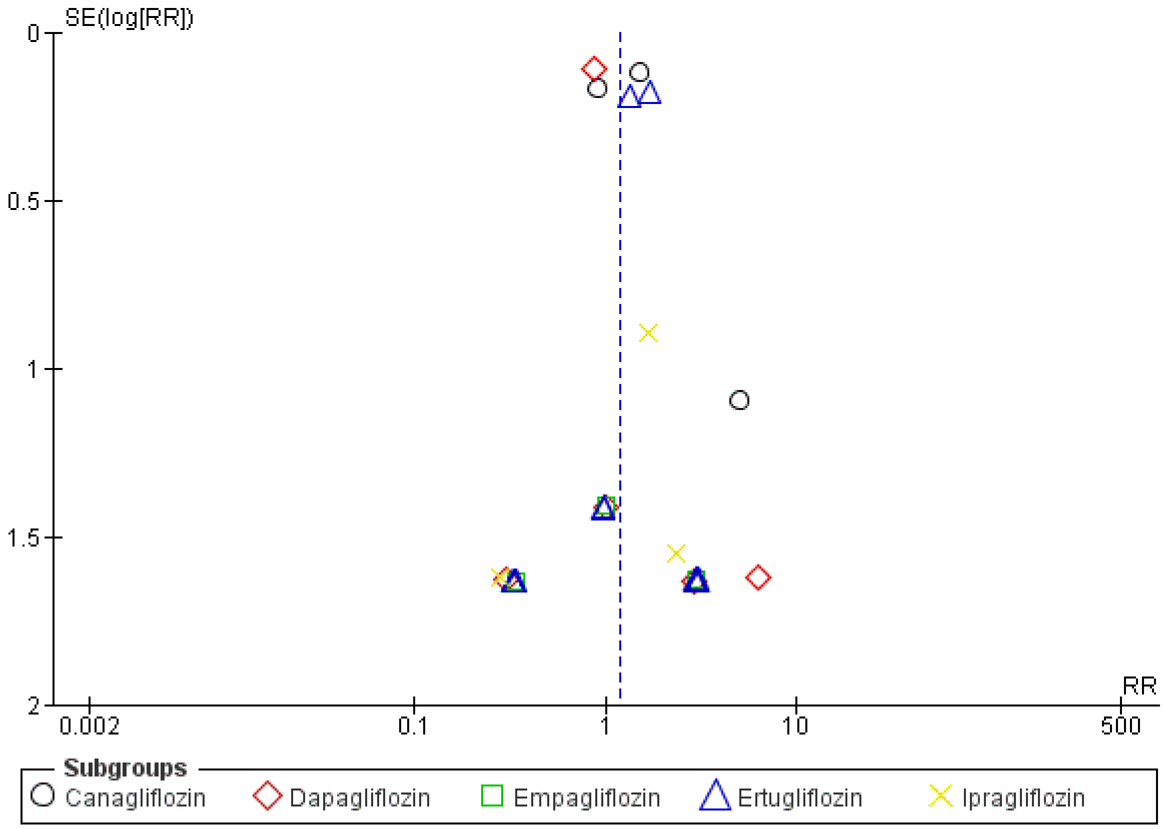

SE, standard error; RR, risk ratio.

Figure S8: Follow-up span sub-analysis for peripheral artery disease.

Study or Subgroup	SGLT2-i Events	SGLT2-i Total	Control Events	Control Total	Weight	Risk Ratio M-H, Random, 95% CI
Cannon 2020 (1)	26	2752	45	2747	19.9%	0.58 [0.36, 0.93]
Cannon 2020 (2)	34	2747	45	2747	20.7%	0.76 [0.49, 1.18]
Katakami 2020	1	169	0	171	1.8%	3.04 [0.12, 73.99]
Neal 2017	79	5795	28	4347	20.9%	2.12 [1.38, 3.25]
Perkovic 2019	14	2202	11	2199	14.2%	1.27 [0.58, 2.79]
Wiviott 2019	62	8582	64	8578	22.4%	0.97 [0.68, 1.37]
**Total (95% CI)**		**22247**		**20789**	**100.0%**	**1.04 [0.67, 1.61]**
Total events	216		193			

Heterogeneity: Tau² = 0.19; Chi² = 19.17, df = 5 (P = 0.002); I² = 74%
Test for overall effect: Z = 0.16 (P = 0.87)

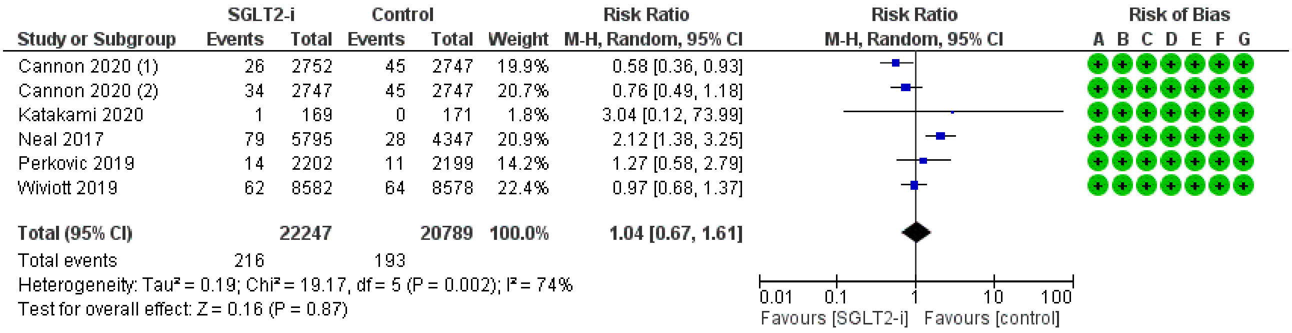

Risk of bias legend
(A) Random sequence generation (selection bias)
(B) Allocation concealment (selection bias)
(C) Blinding of participants and personnel (performance bias)
(D) Blinding of outcome assessment (detection bias)
(E) Incomplete outcome data (attrition bias)
(F) Selective reporting (reporting bias)
(G) Other bias

SGLT2-i, sodium-glucose cotransporter 2 inhibitor(s); M-H, Mantel-Haenszel; CI, confidence interval.

Figure S9: Follow-up span sub-analysis for lower limb ulcers.

	SGLT2-i		Control			Risk Ratio	Risk of Bias						
Study or Subgroup	Events	Total	Events	Total	Weight	M-H, Random, 95% CI	A	B	C	D	E	F	G
Cannon 2020 (1)	12	2752	11	2747	11.5%	1.09 [0.48, 2.46]	+	+	+	+	+	+	+
Cannon 2020 (2)	18	2747	11	2747	13.0%	1.64 [0.77, 3.46]	+	+	+	+	+	+	+
Lingvay 2019	0	394	2	394	1.2%	0.20 [0.01, 4.15]	+	+	+	+	+	+	+
Neal 2017	139	5795	46	4347	27.4%	2.27 [1.63, 3.16]	+	+	+	+	+	+	+
Perkovic 2019	114	2202	87	2199	30.0%	1.31 [1.00, 1.72]	+	+	+	+	+	+	+
Wiviott 2019	22	8582	21	8578	17.0%	1.05 [0.58, 1.90]	+	+	+	+	+	+	+
**Total (95% CI)**		**22472**		**21012**	**100.0%**	**1.44 [1.03, 2.01]**							
Total events	305		178										

Heterogeneity: Tau² = 0.08; Chi² = 10.67, df = 5 (P = 0.06); I² = 53%
Test for overall effect: Z = 2.16 (P = 0.03)

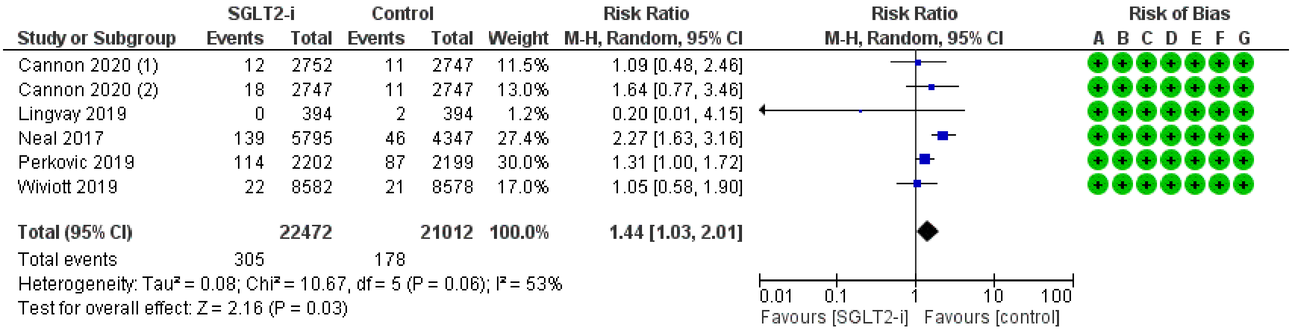

Risk of bias legend
(A) Random sequence generation (selection bias)
(B) Allocation concealment (selection bias)
(C) Blinding of participants and personnel (performance bias)
(D) Blinding of outcome assessment (detection bias)
(E) Incomplete outcome data (attrition bias)
(F) Selective reporting (reporting bias)
(G) Other bias

SGLT2-i, sodium-glucose cotransporter 2 inhibitor(s); M-H, Mantel-Haenszel; CI, confidence interval.

Figure S10: Follow-up span sub-analysis for lower limb fractures.

Study or Subgroup	SGLT2-i Events	SGLT2-i Total	Control Events	Control Total	Weight	Risk Ratio M-H, Random, 95% CI
Barnett 2014	0	37	0	37		Not estimable
Cannon 2020 (1)	23	2752	18	2747	10.2%	1.28 [0.69, 2.36]
Cannon 2020 (2)	20	2747	18	2747	9.6%	1.11 [0.59, 2.10]
Dagogo-Jack 2017 (1)	1	156	1	153	0.5%	0.98 [0.06, 15.54]
Dagogo-Jack 2017 (2)	0	155	1	153	0.4%	0.33 [0.01, 8.02]
Gallo 2019 (1)	0	207	1	209	0.4%	0.34 [0.01, 8.21]
Gallo 2019 (2)	0	205	1	209	0.4%	0.34 [0.01, 8.29]
Hollander 2017 (1)	0	448	1	437	0.4%	0.33 [0.01, 7.96]
Hollander 2017 (2)	2	441	1	437	0.7%	1.98 [0.18, 21.78]
Kawamori 2018	2	182	0	93	0.4%	2.57 [0.12, 52.95]
Lingvay 2019	2	394	1	394	0.7%	2.00 [0.18, 21.97]
Neal 2017	136	5795	85	4347	53.7%	1.20 [0.92, 1.57]
Perkovic 2019	18	2202	12	2199	7.3%	1.50 [0.72, 3.10]
Pratley 2017 (1)	0	250	0	247		Not estimable
Pratley 2017 (2)	0	248	0	247		Not estimable
Pratley 2017 (3)	0	243	0	247		Not estimable
Pratley 2017 (4)	0	245	0	247		Not estimable
Sone 2019 (1)	0	89	1	90	0.4%	0.34 [0.01, 8.16]
Sone 2019 (2)	0	90	1	90	0.4%	0.33 [0.01, 8.08]
Wiviott 2019	30	8582	28	8578	14.6%	1.07 [0.64, 1.79]
**Total (95% CI)**		**25468**		**23908**	**100.0%**	**1.17 [0.96, 1.43]**
Total events	234		170			

Heterogeneity: Tau² = 0.00; Chi² = 4.91, df = 14 (P = 0.99); I² = 0%
Test for overall effect: Z = 1.60 (P = 0.11)

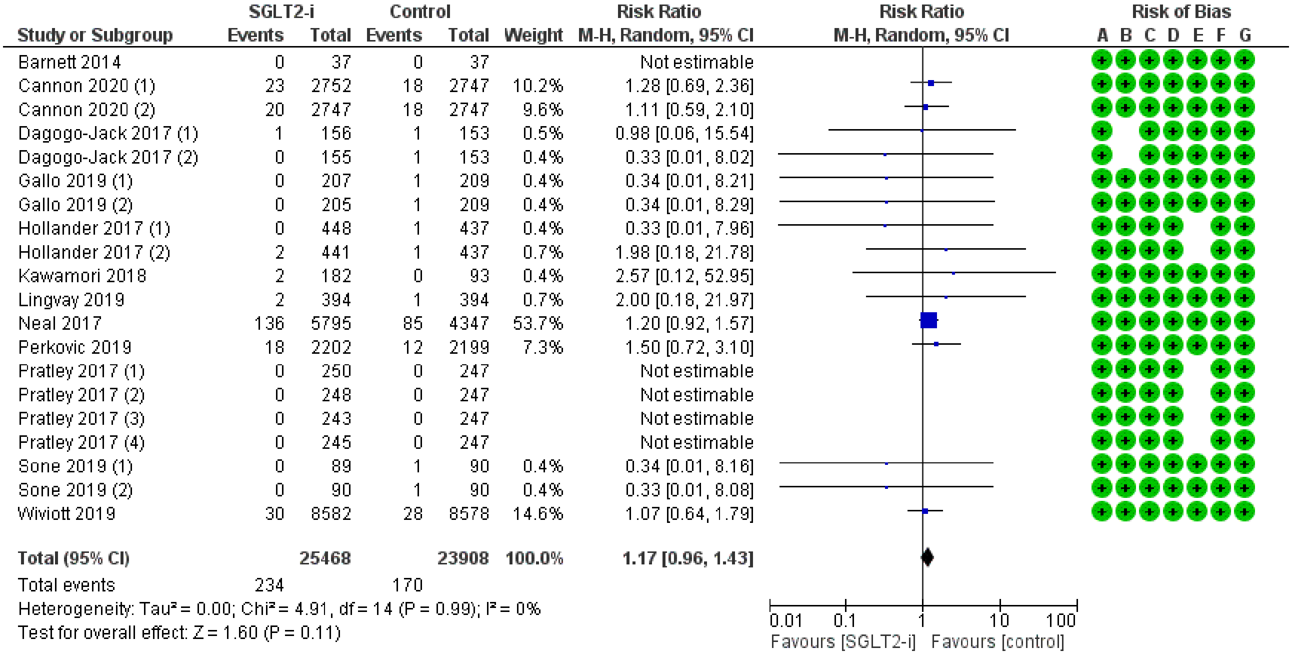

Risk of bias legend
(A) Random sequence generation (selection bias)
(B) Allocation concealment (selection bias)
(C) Blinding of participants and personnel (performance bias)
(D) Blinding of outcome assessment (detection bias)
(E) Incomplete outcome data (attrition bias)
(F) Selective reporting (reporting bias)
(G) Other bias

SGLT2-i, sodium-glucose cotransporter 2 inhibitor(s); M-H, Mantel-Haenszel; CI, confidence interval.

Figure S11: Follow-up span sub-analysis for lower limb amputations.

	SGLT2-i		Control			Risk Ratio
**Study or Subgroup**	**Events**	**Total**	**Events**	**Total**	**Weight**	**M-H, Random, 95% CI**
Cannon 2020 (1)	54	2752	45	2747	14.3%	1.20 [0.81, 1.77]
Cannon 2020 (2)	57	2747	45	2747	14.5%	1.27 [0.86, 1.87]
Gallo 2019 (1)	1	207	0	209	0.4%	3.03 [0.12, 73.92]
Gallo 2019 (2)	2	205	0	209	0.5%	5.10 [0.25, 105.52]
Hollander 2017 (1)	0	448	1	437	0.4%	0.33 [0.01, 7.96]
Hollander 2017 (2)	1	441	1	437	0.6%	0.99 [0.06, 15.79]
Katakami 2020	0	169	0	171		Not estimable
Kawamori 2018	0	182	0	93		Not estimable
Lingvay 2019	0	394	0	394		Not estimable
Neal 2017	210	5795	80	4347	20.0%	1.97 [1.53, 2.54]
Perkovic 2019	70	2202	63	2199	16.5%	1.11 [0.79, 1.55]
Pratley 2017 (1)	0	250	0	247		Not estimable
Pratley 2017 (2)	1	248	0	247	0.4%	2.99 [0.12, 72.99]
Pratley 2017 (3)	0	243	0	247		Not estimable
Pratley 2017 (4)	0	245	0	247		Not estimable
Sone 2019 (1)	0	89	0	90		Not estimable
Sone 2019 (2)	0	90	0	90		Not estimable
Takashima 2018	0	21	0	21		Not estimable
Wheeler 2021	35	1455	38	1451	12.3%	0.92 [0.58, 1.45]
Wiviott 2019	123	8582	113	8578	20.0%	1.09 [0.84, 1.40]
**Total (95% CI)**		**26765**		**25208**	**100.0%**	**1.26 [1.02, 1.56]**
Total events	554		386			

Heterogeneity: $Tau^2 = 0.04$; $Chi^2 = 17.52$, df = 10 (P = 0.06); $I^2 = 43\%$

Test for overall effect: Z = 2.14 (P = 0.03)

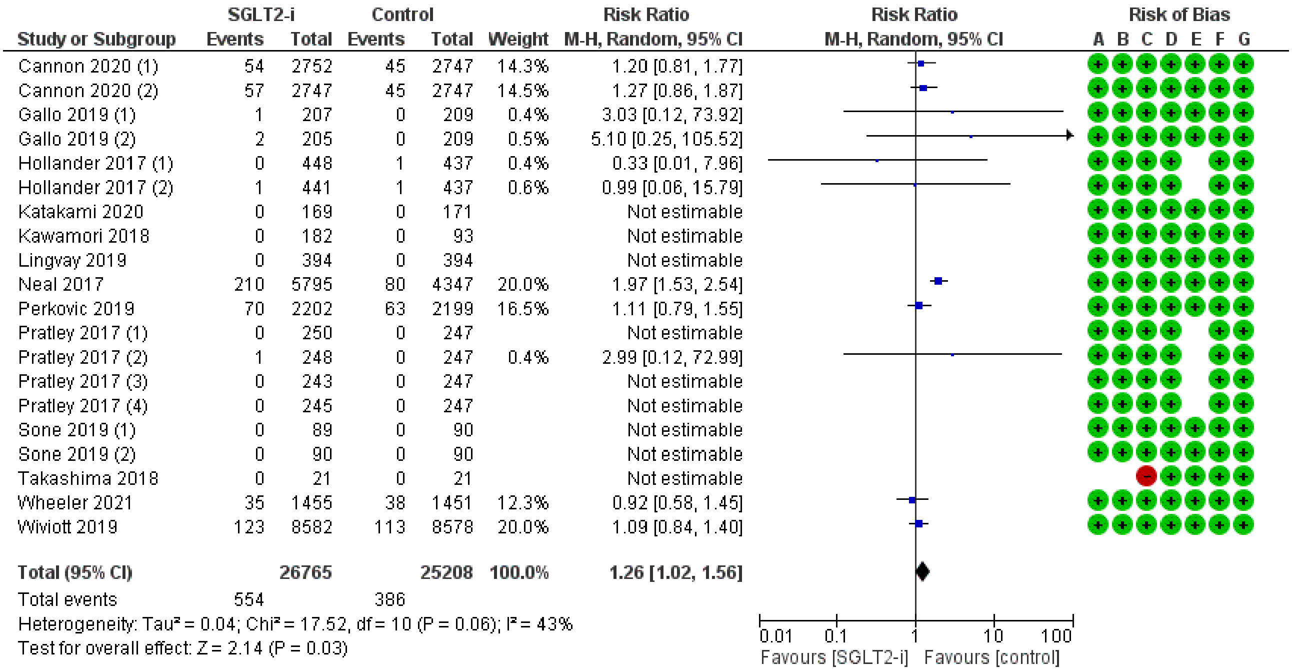

Risk of bias legend

(**A**) Random sequence generation (selection bias)
(**B**) Allocation concealment (selection bias)
(**C**) Blinding of participants and personnel (performance bias)
(**D**) Blinding of outcome assessment (detection bias)
(**E**) Incomplete outcome data (attrition bias)
(**F**) Selective reporting (reporting bias)
(**G**) Other bias

SGLT2-i, sodium-glucose cotransporter 2 inhibitor(s); M-H, Mantel-Haenszel; CI, confidence interval.

Figure S12: Follow-up span sub-analysis for symmetric polyneuropathy.

Study or Subgroup	SGLT2-i Events	SGLT2-i Total	Control Events	Control Total	Weight	Risk Ratio M-H, Random, 95% CI	A	B	C	D	E	F	G
Cannon 2020 (1)	9	2752	3	2747	8.1%	2.99 [0.81, 11.05]	+	+	+	+	+	+	+
Cannon 2020 (2)	1	2747	3	2747	3.2%	0.33 [0.03, 3.20]	+	+	+	+	+	+	+
Lingvay 2019	18	394	9	394	16.2%	2.00 [0.91, 4.40]	+	+	+	+	+	+	+
Neal 2017	134	5795	68	4347	31.6%	1.48 [1.11, 1.97]	+	+	+	+	+	+	+
Perkovic 2019	59	2202	71	2199	29.9%	0.83 [0.59, 1.17]	+	+	+	+	+	+	+
Wiviott 2019	6	8582	8	8578	11.1%	0.75 [0.26, 2.16]	+	+	+	+	+	+	+
**Total (95% CI)**		**22472**		**21012**	**100.0%**	**1.22 [0.80, 1.87]**							
Total events	227		162										

Heterogeneity: Tau² = 0.12; Chi² = 12.01, df = 5 (P = 0.03); I² = 58%
Test for overall effect: Z = 0.94 (P = 0.35)

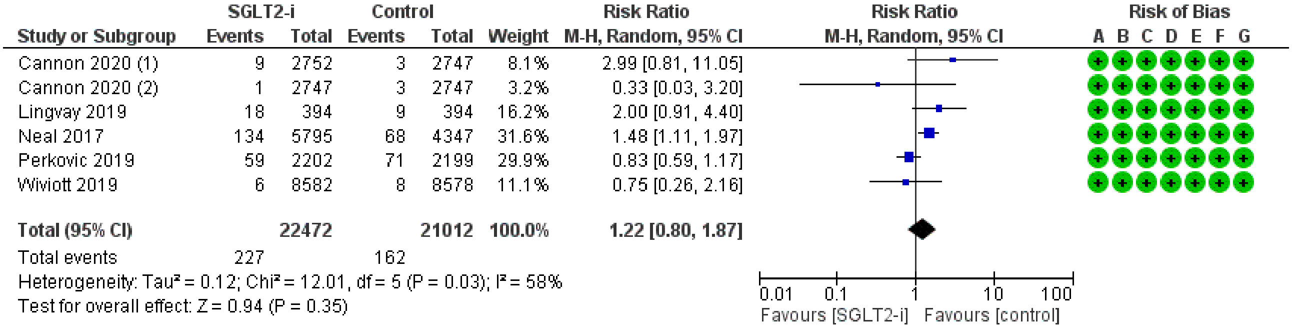

Risk of bias legend
(**A**) Random sequence generation (selection bias)
(**B**) Allocation concealment (selection bias)
(**C**) Blinding of participants and personnel (performance bias)
(**D**) Blinding of outcome assessment (detection bias)
(**E**) Incomplete outcome data (attrition bias)
(**F**) Selective reporting (reporting bias)
(**G**) Other bias

SGLT2-i, sodium-glucose cotransporter 2 inhibitor(s); M-H, Mantel-Haenszel; CI, confidence interval.

Figure S13: Follow-up span sub-analysis for lower limb infections.

Study or Subgroup	SGLT2-i Events	SGLT2-i Total	Control Events	Control Total	Weight	Risk Ratio M-H, Random, 95% CI
Cannon 2020 (1)	63	2752	47	2747	15.8%	1.34 [0.92, 1.94]
Cannon 2020 (2)	80	2747	47	2747	16.5%	1.70 [1.19, 2.43]
Dagogo-Jack 2017 (1)	1	156	1	153	0.6%	0.98 [0.06, 15.54]
Dagogo-Jack 2017 (2)	0	155	1	153	0.5%	0.33 [0.01, 8.02]
Gallo 2019 (1)	1	207	0	209	0.5%	3.03 [0.12, 73.92]
Gallo 2019 (2)	1	205	0	209	0.5%	3.06 [0.13, 74.64]
Hollander 2017 (1)	1	448	1	437	0.6%	0.98 [0.06, 15.55]
Hollander 2017 (2)	0	441	1	437	0.5%	0.33 [0.01, 8.09]
Lingvay 2019	5	394	1	394	1.1%	5.00 [0.59, 42.60]
Neal 2017	201	5795	100	4347	21.5%	1.51 [1.19, 1.91]
Perkovic 2019	64	2202	70	2199	17.4%	0.91 [0.65, 1.27]
Pratley 2017 (1)	0	250	0	247		Not estimable
Pratley 2017 (2)	1	248	0	247	0.5%	2.99 [0.12, 72.99]
Pratley 2017 (3)	0	243	0	247		Not estimable
Pratley 2017 (4)	1	245	0	247	0.5%	3.02 [0.12, 73.88]
Sone 2019 (1)	0	89	1	90	0.5%	0.34 [0.01, 8.16]
Sone 2019 (2)	1	90	1	90	0.6%	1.00 [0.06, 15.74]
Wiviott 2019	154	8582	178	8578	22.4%	0.86 [0.70, 1.07]
**Total (95% CI)**		**25249**		**23778**	**100.0%**	**1.22 [0.97, 1.52]**
Total events	574		449			

Heterogeneity: Tau² = 0.05; Chi² = 23.95, df = 15 (P = 0.07); I² = 37%

Test for overall effect: Z = 1.71 (P = 0.09)

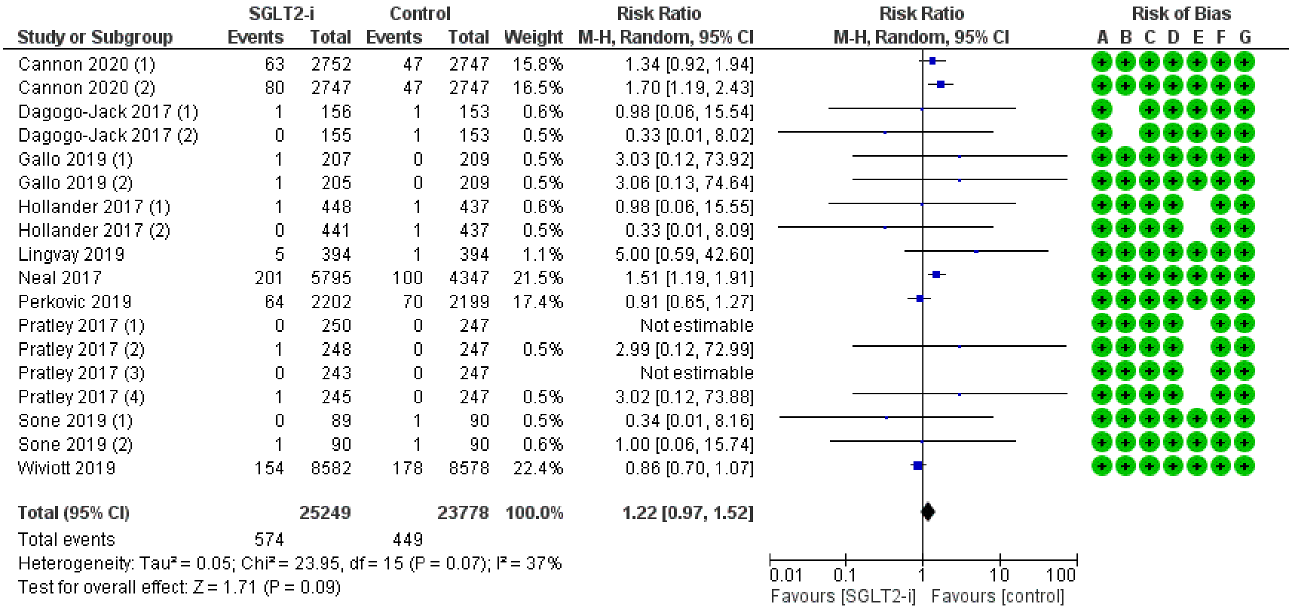

Risk of bias legend

(A) Random sequence generation (selection bias)

(B) Allocation concealment (selection bias)

(C) Blinding of participants and personnel (performance bias)

(D) Blinding of outcome assessment (detection bias)

(E) Incomplete outcome data (attrition bias)

(F) Selective reporting (reporting bias)

(G) Other bias

SGLT2-i, sodium-glucose cotransporter 2 inhibitor(s); M-H, Mantel-Haenszel; CI, confidence interval.

Figure S14: Sensitivity analysis for overall peripheral artery disease.

Study or Subgroup	SGLT2-i Events	SGLT2-i Total	Control Events	Control Total	Weight	Risk Ratio M-H, Random, 95% CI
**1.12.1 Canagliflozin**						
Neal 2017	79	5795	28	4347	0.0%	2.12 [1.38, 3.25]
Perkovic 2019	14	2202	11	2199	10.4%	1.27 [0.58, 2.79]
**Subtotal (95% CI)**		**2202**		**2199**	**10.4%**	**1.27 [0.58, 2.79]**
Total events	14		11			
Heterogeneity: Not applicable						
Test for overall effect: Z = 0.60 (P = 0.55)						
**1.12.2 Dapagliflozin**						
Schumm-Draeger 2014 (2)	0	199	0	101		Not estimable
Wiviott 2019	62	8582	64	8578	37.9%	0.97 [0.68, 1.37]
**Subtotal (95% CI)**		**8781**		**8679**	**37.9%**	**0.97 [0.68, 1.37]**
Total events	62		64			
Heterogeneity: Not applicable						
Test for overall effect: Z = 0.18 (P = 0.86)						
**1.12.3 Ertugliflozin**						
Cannon 2020 (1)	26	2752	45	2747	23.9%	0.58 [0.36, 0.93]
Cannon 2020 (2)	34	2747	45	2747	27.1%	0.76 [0.49, 1.18]
**Subtotal (95% CI)**		**5499**		**5494**	**51.1%**	**0.67 [0.48, 0.92]**
Total events	60		90			
Heterogeneity: Tau² = 0.00; Chi² = 0.66, df = 1 (P = 0.42); I² = 0%						
Test for overall effect: Z = 2.44 (P = 0.01)						
**1.12.4 Tofogliflozin**						
Katakami 2020	1	169	0	171	0.7%	3.04 [0.12, 73.99]
**Subtotal (95% CI)**		**169**		**171**	**0.7%**	**3.04 [0.12, 73.99]**
Total events	1		0			
Heterogeneity: Not applicable						
Test for overall effect: Z = 0.68 (P = 0.50)						
**Total (95% CI)**		**16651**		**16543**	**100.0%**	**0.83 [0.64, 1.08]**
Total events	137		165			
Heterogeneity: Tau² = 0.02; Chi² = 4.90, df = 4 (P = 0.30); I² = 18%						
Test for overall effect: Z = 1.38 (P = 0.17)						
Test for subgroup differences: Chi² = 4.24, df = 3 (P = 0.24), I² = 29.2%						

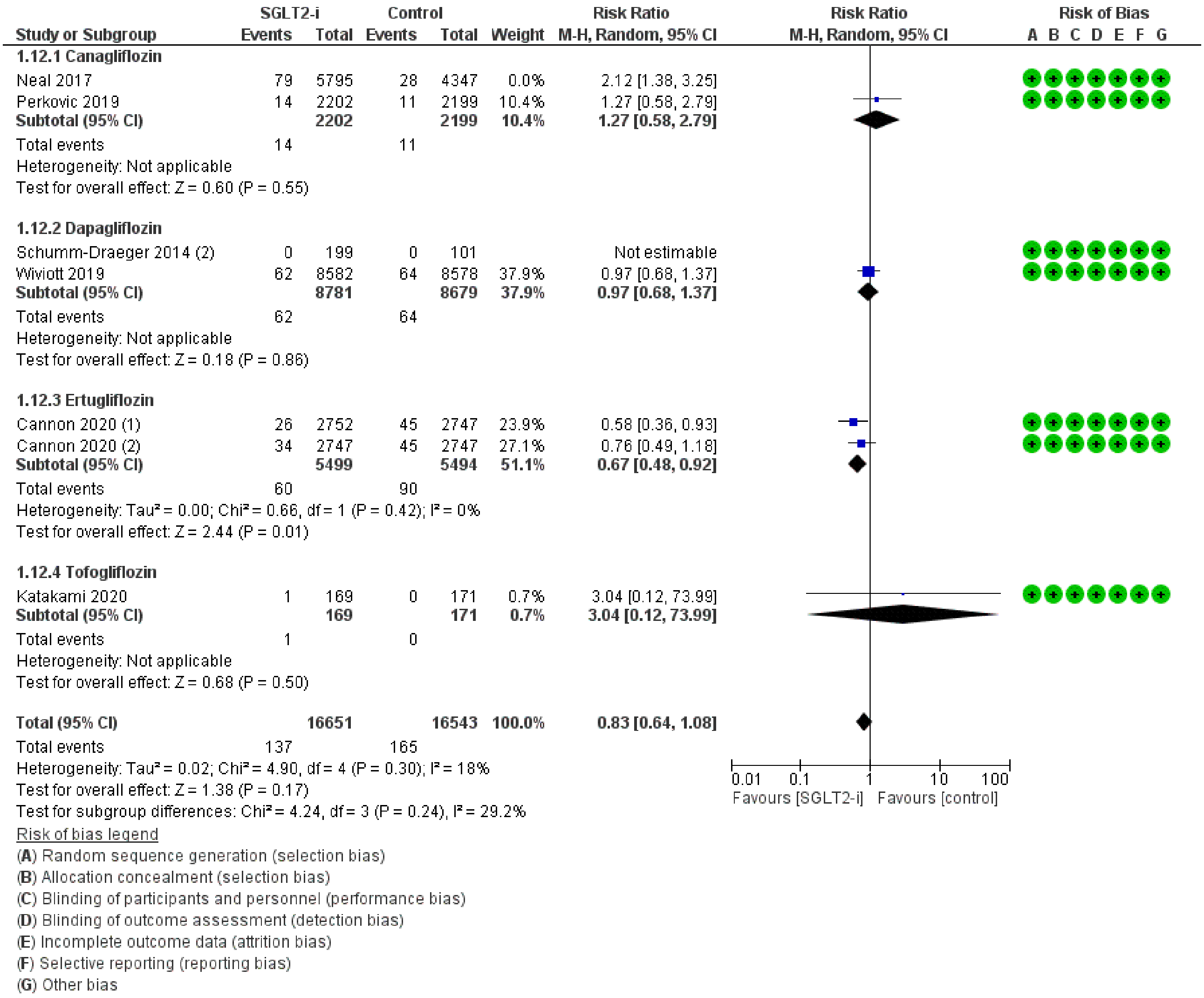

Risk of bias legend
(A) Random sequence generation (selection bias)
(B) Allocation concealment (selection bias)
(C) Blinding of participants and personnel (performance bias)
(D) Blinding of outcome assessment (detection bias)
(E) Incomplete outcome data (attrition bias)
(F) Selective reporting (reporting bias)
(G) Other bias

SGLT2-i, sodium-glucose cotransporter 2 inhibitor(s); M-H, Mantel-Haenszel; CI, confidence interval. By solely excluding Neal 2017, I^2 drops from 74% to 18%, while the pooled risk ratio remains nonsignificant; global heterogeneity stays high otherwise. There is no clear evidence of strong clinical variability nor pervasive biases in support.

Figure S15: Sensitivity analysis for peripheral artery disease with a follow-up ≥ 52 weeks.

	SGLT2-i		Control			Risk Ratio
**Study or Subgroup**	**Events**	**Total**	**Events**	**Total**	**Weight**	**M-H, Random, 95% CI**
Cannon 2020 (1)	26	2752	45	2747	23.9%	0.58 [0.36, 0.93]
Cannon 2020 (2)	34	2747	45	2747	27.1%	0.76 [0.49, 1.18]
Katakami 2020	1	169	0	171	0.7%	3.04 [0.12, 73.99]
Neal 2017	79	5795	28	4347	0.0%	2.12 [1.38, 3.25]
Perkovic 2019	14	2202	11	2199	10.4%	1.27 [0.58, 2.79]
Wiviott 2019	62	8582	64	8578	37.9%	0.97 [0.68, 1.37]
**Total (95% CI)**		**16452**		**16442**	**100.0%**	**0.83 [0.64, 1.08]**
Total events	137		165			

Heterogeneity: Tau² = 0.02; Chi² = 4.90, df = 4 (P = 0.30); I² = 18%
Test for overall effect: Z = 1.38 (P = 0.17)

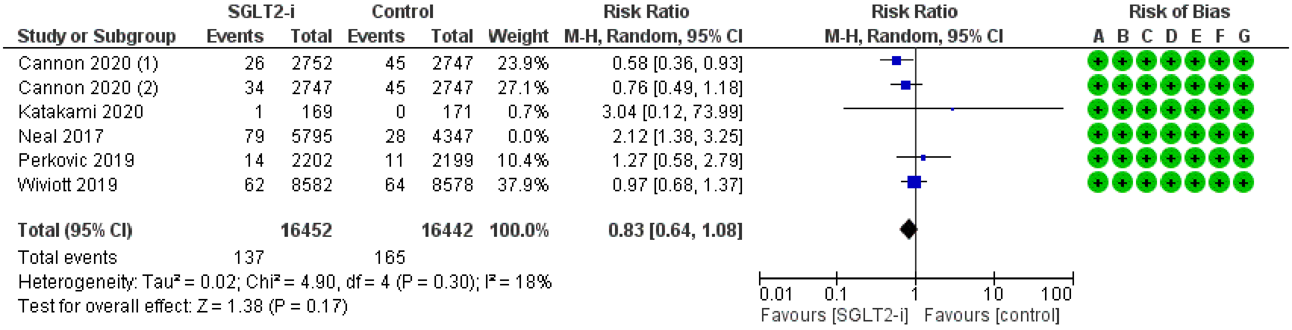

Risk of bias legend
(**A**) Random sequence generation (selection bias)
(**B**) Allocation concealment (selection bias)
(**C**) Blinding of participants and personnel (performance bias)
(**D**) Blinding of outcome assessment (detection bias)
(**E**) Incomplete outcome data (attrition bias)
(**F**) Selective reporting (reporting bias)
(**G**) Other bias

SGLT2-i, sodium-glucose cotransporter 2 inhibitor(s); M-H, Mantel-Haenszel; CI, confidence interval. By solely excluding Neal 2017, I^2 drops from 74% to 18%, while the pooled risk ratio remains nonsignificant; global heterogeneity stays high otherwise. There is no clear evidence of strong clinical variability nor pervasive biases in support.

Figure S16: Sensitivity analysis for lower limb ulcers with a follow-up ≥ 52 weeks.

Study or Subgroup	SGLT2-i Events	SGLT2-i Total	Control Events	Control Total	Weight	Risk Ratio M-H, Random, 95% CI	A	B	C	D	E	F	G
Cannon 2020 (1)	12	2752	11	2747	7.6%	1.09 [0.48, 2.46]	+	+	+	+	+	+	+
Cannon 2020 (2)	18	2747	11	2747	9.1%	1.64 [0.77, 3.46]	+	+	+	+	+	+	+
Lingvay 2019	0	394	2	394	0.6%	0.20 [0.01, 4.15]	+	+	+	+	+	+	+
Neal 2017	139	5795	46	4347	0.0%	2.27 [1.63, 3.16]	+	+	+	+	+	+	+
Perkovic 2019	114	2202	87	2199	68.4%	1.31 [1.00, 1.72]	+	+	+	+	+	+	+
Wiviott 2019	22	8582	21	8578	14.3%	1.05 [0.58, 1.90]	+	+	+	+	+	+	+
**Total (95% CI)**		**16677**		**16665**	**100.0%**	**1.26 [1.01, 1.58]**							
Total events	166		132										

Heterogeneity: Tau² = 0.00; Chi² = 2.45, df = 4 (P = 0.65); I² = 0%
Test for overall effect: Z = 2.02 (P = 0.04)

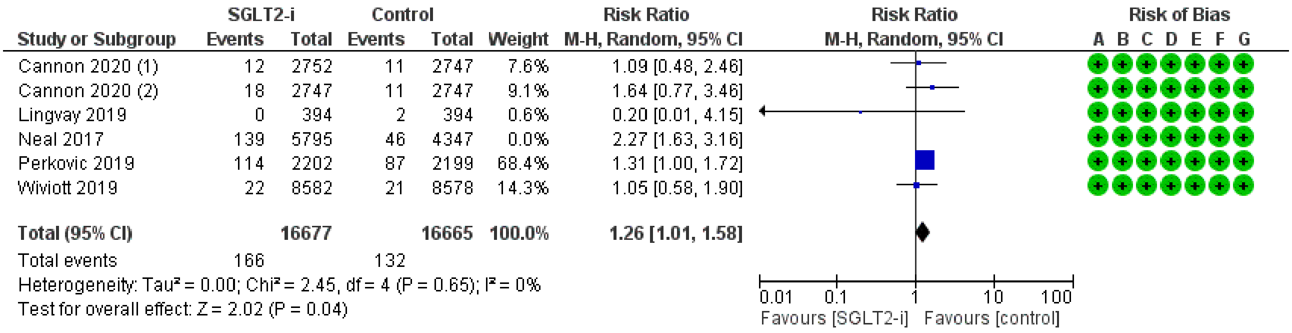

Risk of bias legend
(**A**) Random sequence generation (selection bias)
(**B**) Allocation concealment (selection bias)
(**C**) Blinding of participants and personnel (performance bias)
(**D**) Blinding of outcome assessment (detection bias)
(**E**) Incomplete outcome data (attrition bias)
(**F**) Selective reporting (reporting bias)
(**G**) Other bias

SGLT2-i, sodium-glucose cotransporter 2 inhibitor(s); M-H, Mantel-Haenszel; CI, confidence interval. By solely excluding Neal 2017, I^2 drops from 53% to 0%, while the pooled risk ratio remains nonsignificant; global heterogeneity stays high otherwise. There is no clear evidence of strong clinical variability nor pervasive biases in support.

Figure S17: Sensitivity analysis for symmetric polyneuropathy with a follow-up ≥ 52 weeks.

Study or Subgroup	SGLT2-i Events	SGLT2-i Total	Control Events	Control Total	Weight	Risk Ratio M-H, Random, 95% CI	Risk of Bias A	B	C	D	E	F	G
Cannon 2020 (1)	9	2752	3	2747	7.9%	2.99 [0.81, 11.05]	+	+	+	+	+	+	+
Cannon 2020 (2)	1	2747	3	2747	2.8%	0.33 [0.03, 3.20]	+	+	+	+	+	+	+
Lingvay 2019	18	394	9	394	18.8%	2.00 [0.91, 4.40]	+	+	+	+	+	+	+
Neal 2017	134	5795	68	4347	59.1%	1.48 [1.11, 1.97]	+	+	+	+	+	+	+
Perkovic 2019	59	2202	71	2199	0.0%	0.83 [0.59, 1.17]	+	+	+	+	+	+	+
Wiviott 2019	6	8582	8	8578	11.5%	0.75 [0.26, 2.16]	+	+	+	+	+	+	+
**Total (95% CI)**		**20270**		**18813**	**100.0%**	**1.47 [1.00, 2.16]**							
Total events	168		91										

Heterogeneity: Tau² = 0.04; Chi² = 4.94, df = 4 (P = 0.29); I² = 19%
Test for overall effect: Z = 1.96 (P = 0.05)

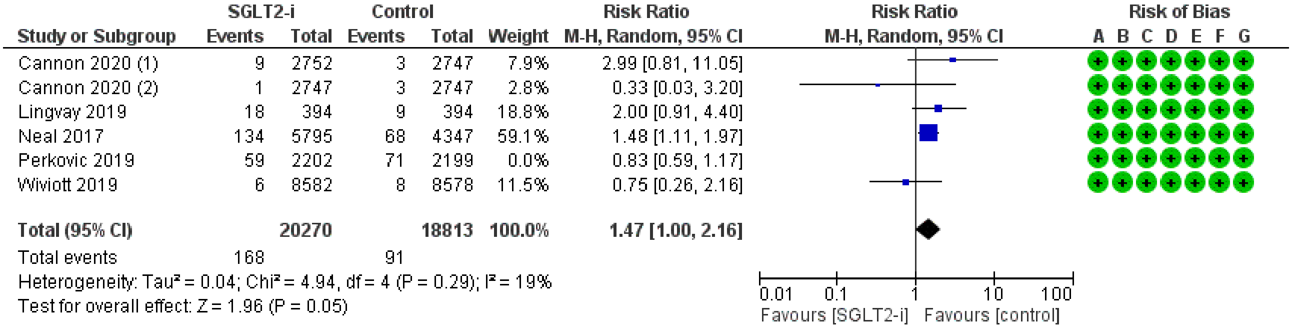

Risk of bias legend
(**A**) Random sequence generation (selection bias)
(**B**) Allocation concealment (selection bias)
(**C**) Blinding of participants and personnel (performance bias)
(**D**) Blinding of outcome assessment (detection bias)
(**E**) Incomplete outcome data (attrition bias)
(**F**) Selective reporting (reporting bias)
(**G**) Other bias

SGLT2-i, sodium-glucose cotransporter 2 inhibitor(s); M-H, Mantel-Haenszel; CI, confidence interval. By solely excluding Perkovic 2019, I^2 drops from 58% to 19%, while the pooled risk ratio remains nonsignificant; global heterogeneity stays high otherwise. There is no clear evidence of strong clinical variability nor pervasive biases in support.

Table S1: Database query strings and filters.

MEDLINE (PubMed)
Search: "type 2 diabetes mellitus AND (canagliflozin OR dapagliflozin OR empagliflozin OR ertugliflozin OR ipragliflozin OR luseogliflozin OR tofogliflozin)". Filters: Full text; Randomized Controlled Trial; Humans; English; Adult: 19+ years; Young Adult: 19-24 years; Adult: 19-44 years; Middle Aged + Aged: 45+ years; Middle Aged: 45-64 years; Aged: 65+ years; 80 and over: 80+ years.
**Embase**
Search: "type AND ('2'/exp OR 2) AND ('diabetes'/exp OR diabetes) AND mellitus AND ('canagliflozin'/exp OR canagliflozin OR 'dapagliflozin'/exp OR dapagliflozin OR 'empagliflozin'/exp OR empagliflozin OR 'ertugliflozin'/exp OR ertugliflozin OR 'ipragliflozin'/exp OR ipragliflozin OR 'luseogliflozin'/exp OR luseogliflozin OR 'tofogliflozin'/exp OR tofogliflozin) AND [randomized controlled trial]/lim AND [english]/lim AND ([adult]/lim OR [young adult]/lim OR [middle aged]/lim OR [aged]/lim OR [very elderly]/lim) AND [humans]/lim AND ([embase]/lim OR [medline]/lim)".
**Cochrane Central Register of Controlled Trials (CENTRAL)**
Search: "type 2 diabetes mellitus AND (canagliflozin OR dapagliflozin OR empagliflozin OR ertugliflozin OR ipragliflozin OR luseogliflozin OR tofogliflozin)". Fields: Title; Abstract; Keyword.

Table S2: MedDRA terms for peripheral artery disease.

**Peripheral artery disease**
Femoral artery occlusion; Iliac artery occlusion; Iliac artery stenosis; Intermittent claudication; Leriche syndrome; Peripheral arterial occlusive disease; Peripheral artery occlusion; Peripheral artery stenosis

Table S3: MedDRA terms for lower limb ulcers.

**Lower limb ulcers**
Diabetic ulcer; Extremity necrosis; Ischaemic necrosis; Ischaemic skin ulcer; Necrosis; Neuropathic ulcer; Skin necrosis; Skin ulcer

Table S4. MedDRA terms for symmetric polyneuropathy.

**Symmetric polyneuropathy**
Diabetic neuropathy; Peripheral neuropathy; Peripheral sensorimotor neuropathy; Polyneuropathy

Table S5: MedDRA terms for lower limb infections.

**Lower limb infections**
Acute osteomyelitis; Arteriosclerotic gangrene; Cellulitis; Chronic osteomyelitis; Diabetic foot infection; Diabetic gangrene; Erysipelas; Gangrene; Gangrenous cellulitis; Gas gangrene; Infected skin ulcer; Limb abscess; Necrotising fasciitis; Septic arthritis; Skin abscess; Staphylococcal cellulitis; Subcutaneous abscess; Tinea pedis; Toe gangrene

Table S6: Baseline characteristics.

	SGLT2-i	Control
N	29,491	23,052
Age (years) – mean ± SD	59.3 ± 4.4	58.9 ± 4.3
HbA1c (mmol/mol) – mean ± SD	64.8 ± 6.6	65.0 ± 6.5
BMI (kg/m^2) – mean ± SD	29.7 ± 2.7	29.6 ± 3.0
**Overall population**		
Male – % (min-max)	59.8 (40.3-78.0)	
White – % (min-max)	44.5 (0-93.3)	
Black – % (min-max)	2.8 (0-10.1)	
Asian – % (min-max)	49.2 (0-100)	
Other – % (min-max)	3.5 (0-12.3)	
Follow-up span – weeks (min-max)	50.9 (12-219)	

N, number; SD, standard deviation.

Table S7: GRADE summary.

Certainty assessment							№ of patients		Effect		Certainty	Importance
№ of studies	Study design	Risk of bias	Inconsistency	Indirectness	Imprecision	Other considerations	SGLT2-i	Control	Relative (95% CI)	Absolute (95% CI)		
**Osteomyelitis**												
12	randomised trials	not serious	not serious	not serious	serious[a]	none	79/23,521 (0.3%)	71/21,974 (0.3%)	RR 1.04 (0.76 to 1.44)	0 fewer per 1,000 (from 1 fewer to 1 more)	⊕⊕⊕◯ Moderate	CRITICAL
**PAD**												
7	randomised trials	not serious	not serious	not serious	not serious	none	216/22,446 (1.0%)	193/20,890 (0.9%)	RR 1.04 (0.67 to 1.61)	0 fewer per 1,000 (from 3 fewer to 6 more)	⊕⊕⊕⊕ High	CRITICAL
**Lower limb ulcers**												
9	randomised trials	not serious	not serious	not serious	not serious	none	305/22,559 (1.4%)	181/21,100 (0.9%)	RR 1.39 (1.01 to 1.91)	3 more per 1,000 (from 0 fewer to 8 more)	⊕⊕⊕⊕ High	CRITICAL
**Lower limb fractures**												
33	randomised trials	not serious	not serious	not serious	not serious	none	237/26,821 (0.9%)	177/25,143 (0.7%)	RR 1.11 (0.95 to 1.40)	1 more per 1,000 (from 0 fewer to 3 more)	⊕⊕⊕⊕ High	CRITICAL
**Amputations**												
34	randomised trials	not serious	not serious	not serious	not serious	none	556/27,686 (2.0%)	386/26,117 (1.5%)	RR 1.27 (1.04 to 1.55)	4 more per 1,000 (from 1 more to 8 more)	⊕⊕⊕⊕ High	CRITICAL
**Symmetric polyneuropathy**												

Certainty assessment							№ of patients		Effect		Certainty	Importance
**№ of studies**	**Study design**	**Risk of bias**	**Inconsistency**	**Indirectness**	**Imprecision**	**Other considerations**	**SGLT2-i**	**Control**	**Relative (95% CI)**	**Absolute (95% CI)**		
11	randomised trials	not serious	not serious	not serious	not serious	none	229/22,758 (1.0%)	166/21,286 (0.8%)	RR 1.17 (0.82 to 1.63)	1 more per 1,000 (from 1 fewer to 5 more)	⊕⊕⊕⊕ High	CRITICAL
**Lower limb infections**												
31	randomised trials	not serious	not serious	not serious	not serious	none	585/26,415 (2.2%)	456/24,871 (1.8%)	RR 1.20 (1.02 to 1.40)	4 more per 1,000 (from 0 fewer to 7 more)	⊕⊕⊕⊕ High	CRITICAL

№, number; SGLT2-i, sodium-glucose cotransporter 2 inhibitor(s); CI, confidence interval; RR, risk ratio. [1]Serious imprecision due to a low number of events.
